# A Low-Cost Library Construction Protocol and Data Analysis Pipeline for Illumina-Based Strand-Specific Multiplex RNA-Seq

**DOI:** 10.1371/journal.pone.0026426

**Published:** 2011-10-19

**Authors:** Lin Wang, Yaqing Si, Lauren K. Dedow, Ying Shao, Peng Liu, Thomas P. Brutnell

**Affiliations:** 1 Boyce Thompson Institute for Plant Research, Cornell University, Ithaca, New York, United States of America; 2 Department of Statistics, Iowa State University, Ames, Iowa, United States of America; 3 Department of Microbiology and Immunology, Weill Cornell Medical College, New York, New York, United States of America; University of Illinois, United States of America

## Abstract

The emergence of NextGen sequencing technology has generated much interest in the exploration of transcriptomes. Currently, Illumina Inc. (San Diego, CA) provides one of the most widely utilized sequencing platforms for gene expression analysis. While Illumina reagents and protocols perform adequately in RNA-sequencing (RNA-seq), alternative reagents and protocols promise a higher throughput at a much lower cost. We have developed a low-cost and robust protocol to produce Illumina-compatible (GAIIx and HiSeq2000 platforms) RNA-seq libraries by combining several recent improvements. First, we designed balanced adapter sequences for multiplexing of samples; second, dUTP incorporation in 2^nd^ strand synthesis was used to enforce strand-specificity; third, we simplified RNA purification, fragmentation and library size-selection steps thus drastically reducing the time and increasing throughput of library construction; fourth, we included an RNA spike-in control for validation and normalization purposes. To streamline informatics analysis for the community, we established a pipeline within the iPlant Collaborative. These scripts are easily customized to meet specific research needs and improve on existing informatics and statistical treatments of RNA-seq data. In particular, we apply significance tests for determining differential gene expression and intron retention events. To demonstrate the potential of both the library-construction protocol and data-analysis pipeline, we characterized the transcriptome of the rice leaf. Our data supports novel gene models and can be used to improve current rice genome annotation. Additionally, using the rice transcriptome data, we compared different methods of calculating gene expression and discuss the advantages of a strand-specific approach to detect bona-fide anti-sense transcripts and to detect intron retention events. Our results demonstrate the potential of this low cost and robust method for RNA-seq library construction and data analysis.

## Introduction

The advent of ultra-high-throughput sequencing (UHTS) technology has invoked a paradigm shift in the field of genomics and transcriptomics [1], [2]. It is now possible to obtain whole-genome scale information at a highly accelerated rate. This advancement in sequencing technology has led to new opportunities to explore global genomic and transcriptomic landscapes; such studies include whole-genome de novo/re-sequencing [3], [4], bisulfite-sequencing [5], [6], chromatin immuno-precipitation-sequencing (Chip-seq) [7], [8], and RNA sequencing (RNA-seq) [9], [10]. Together, these newly developed technologies provide new insight into biological systems. In particular, RNA-seq provides highly resolved gene expression data, enables the identification of alternatively spliced transcripts and facilitates gene discovery through annotation improvements. When compared to the standard platform of transcriptomics, microarray analysis, RNA-seq provides orders of magnitude increased throughput for a comparable cost, increased sensitivity and superior resolution and accuracy for expression profiling experiments [2].

Much of the advantage and added functionality of RNA-seq over microarray analysis lies in the methodology of transcript detection. Microarray analysis uses an indirect hybridization-based detection method where a population of pre-synthesized and immobilized nucleotides serve as probes to monitor gene expression through fluorescence signals. Gene expression values are calculated from the fluorescence intensity or a ratio of the intensities. In contrast, RNA-seq uses direct sequence-based detection to quantify gene expression. Since no pre-determined probes are used, RNA-seq is considered an open platform, as no previous annotation of the target genome is needed. The dynamic range of gene expression derived from RNA-seq is much higher relative to microarray analysis, and is largely due to the fact gene quantification is performed by simply counting reads, while fluorescent intensity is usually constrained by a saturation ceiling as an innate property of the probes or the instruments used to detect the signal. For instance, in leaf sections photosynthesis-related genes are so highly expressed that they constitute over 30% of the total transcriptome [11] and can easily saturate detection limits in microarray analysis [12].

Despite the many advantages RNA-seq offers, it is still a relatively new methodology with developments continuing for both experimental procedures and subsequent data analyses. For instance, strand-specific RNA-seq protocols been developed [13], but they have not yet been widely adapted by the community. As one may expect from such a rapidly evolving field, no official strand-specific RNA-seq pipeline is yet publicly available from Illumina and only few are available from other companies (e.g. Epicenter). Additionally, no standard has been established for methods of processing RNA-seq data for gene expression estimation, normalization, comparison and experimental design. A few noteworthy computational pipelines are currently being developed, however, that are gaining community acceptance, these include the “Tuxedo package” - Bowtie, Tophat and Cufflinks [14], [15], [16] that align RNA-seq reads to the genome, determine and align reads to splice junctions and calculate FPKM/RPKM (fragments/reads per kilobase of exon per million fragments/reads mapped) - a normalized value representing gene expression [17]. The Burrows-Wheeler Aligner (BWA) that was designed to quickly align reads to a genome, allowing gaps or deletions [18], is also widely utilized, but lacks the ability to align reads to splice junctions. Supersplat is another software program that aligns RNA-seq reads to the genome and splice junctions but does not support strand-specific protocols [19].

Although RNA-seq has a relatively short history, it has quickly become the method of choice for analyzing transcriptomes. However, one obstacle that hinders broad acceptance of RNA-seq is cost. While UHTS technologies have decreased the overall cost of sequencing on a base pair basis, the direct cost remains inhibitory for many laboratories to perform a large number of UHTS-based experiments. The current dominate RNA-seq platform provider Illumina (www.illumina.com) [1] and its latest sequencing machine - Hiseq2000 (first commercialized during mid-2010) is capable of producing approximately 200 million clusters per lane that typically yield 180–190 million sequencing reads post filtering (TruSeq Cluster Kit v3). When considering the cost of reagents for library construction, costs/sample can run close to $50 with additional costs for sequencing. Thus, cost remains a serious limitation to broader application of RNAseq technology in high-throughput applications.

In this report, we describe a low-cost and robust method of generating strand-specific Illumina-compatible libraries for RNA-seq and a data analysis pipeline to improve gene quantification and detection. By using the alternative reagents and protocols, we are able to reduce the cost to approximately $5 per library. We designed a series of expandable multiplex adaptors that permit pooling of multiple samples into one lane of an Illumina flowcell to reduce sequencing costs and improve experimental design. We also incorporate an aRNA spike-in control to validate library construction and sequencing and as an optional method for normalization. The experimental protocol was streamlined so that over 32 samples can be constructed in less than two days by a single researcher. Using this custom protocol and computational pipeline, we analyzed the rice leaf transcriptome. We detect previously un-annotated genes, improve existing gene models and map novel anti-sense transcripts. We also compare methods of normalization for calculating gene expression and describe a novel statistical approach to detect intron retention events. In summary, our method and data analysis pipeline substantially improve both library construction and data analysis, providing the RNA-seq community with an accessible, easily adaptable and robust tools for transcriptomics studies.

## Results and Discussion

### Overview of the library construction protocol

**Figure 1:**
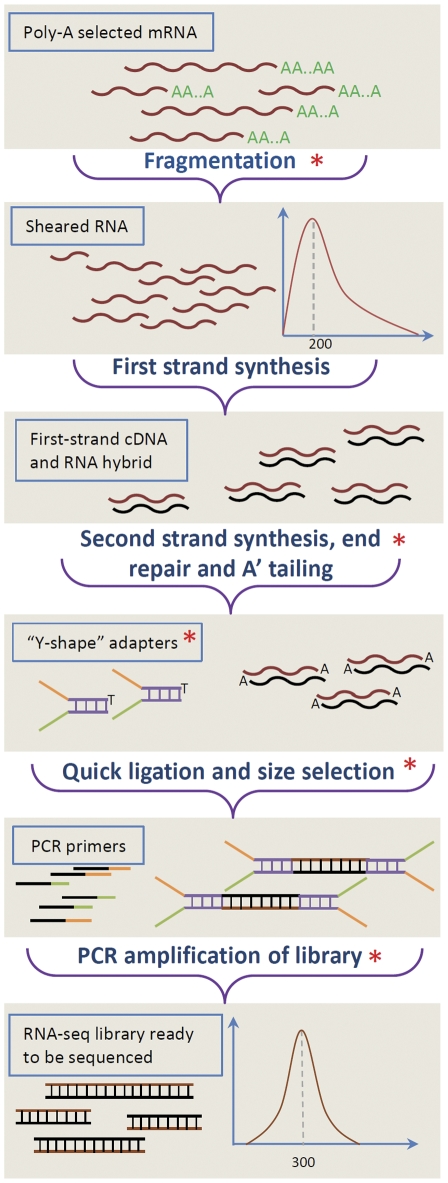
Overview of RNA-seq library construction. mRNA is purified from total RNA and fragmented to the desired size range. Next, the sheared RNA is reverse-transcribed to cDNA to form a DNA/RNA hybrid. The double-stranded cDNA is then synthesized, end-repaired and adenylated. Illumina adaptors are ligated to the processed double-stranded DNA and size selected. Finally, the size-selected ligated DNA products are amplified using primers to produce a sequence-ready library.

**Figure 2:**
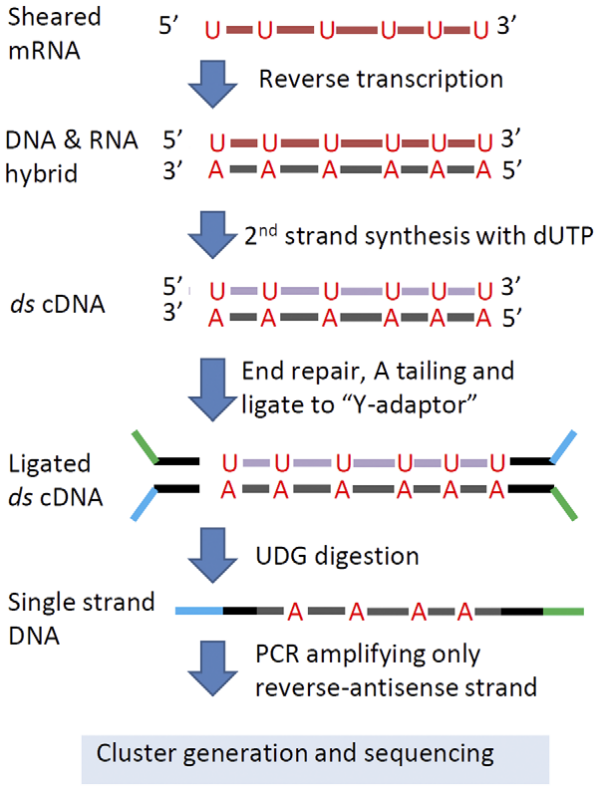
Enforcing strand specificity using dUTP. dTTP is substituted with dUTP during second strand cDNA synthesis. Y-shaped (partial-complementary) adapters are ligated and the dUTP-marked strand is digested with uracil-DNA gylcosylase (UDG). PCR amplification of this single strand confers strand specificity.

The primary workflow of our improved RNA-seq library construction protocol does not diverge significantly from the standard Illumina library construction procedures illustrated in Figure 1. However, we have implemented a number of key improvements at steps marked with red asterisks (Fig. 1). Most importantly, we incorporated a step that preserves the strand-specific nature of mRNA molecules. In addition, we incorporated aRNA spike-in controls added to each RNA input before fragmentation. The aRNA spike-in controls are synthesized *in vitro* from four distinct human cDNA sources that have no homology to plant species (e.g. maize, rice, Arabidopsis, Setaria, Brachypodium, Barley, potato and tomato). The added aRNA spike-in control was used to validate sequencing results and provided an alternative parameter for normalization. However, as shown in Figure S1, spike-in based normalization underperforms when compared to other methods of normalization. The principle of how strand-specific information is retained is illustrated in Figure 2 and is an adaptation of a robust technique where the second-strand cDNA is marked with deoxyuridine triphosphate (dUTP) in place of deoxythymidine triphosphate (dTTP) [13], [21]. We also simplified the fragmentation procedure for the RNA input: instead of using a specific fragmentation buffer, we opted to use reverse transcription (RT) first-strand buffer (Invitrogen, CA) directly, which eliminated the need to purify fragmented RNA. The average size of the RT-buffer fragmented RNA is approximately 200 bps with a 5 minute treatment at 94 degrees as measured by the Agilent Bioanalyzer (Figure S2), which is the suggested size distribution for RNA-seq library on Illumina platform.

The other important enhancement in our protocol is the simplification of purification steps during library construction. Purification is particularly important for the dUTP-based strand-specific protocol to ensure there is no carryover of dTTP after first strand synthesis that may compromise the strand-specificity of the final library. For all the purification steps, we used SPRI (Solid Phase Reversible Immobilization) paramagnetic beads (Beckman Coulter, Danvers, MA), which reduces the cost and time needed for purification, as it is no longer necessary to use nucleotide purification columns (e.g. Qiagen RNA/DNA purification columns). Furthermore, using the SPRI beads, we developed a novel method to obtain the desired size distribution of fragmented RNA by replacing the stock buffer of the SPRI bead suspension with varying concentrations of polyethylene glycerol m.w. 8000 (PEG-8000). Our approach replaces the time-consuming gel-based size-selection method, and improves yield of purified fragments. Figure S3 demonstrates the use of different PEG-8000 concentrations to size fractionate RNA and the corresponding library size distribution analyzed by ImageJ (http://rsbweb.nih.gov/ij/). It is known that variation in library size affects how clusters are generated on the Illumina flowcell and base-calling quality. When the size distribution is large, fewer clusters are generated that are suitable for base-calling (www.illumina.com). When the library is of desired uniform size, more clusters can be generated on a flow cell to produce a greater number of high quality reads. Using our approach, the library size distribution can be controlled (e.g. the GAIIx and HiSeq2000 platforms have different optimal library size requirements).

An important consideration when designing RNA-seq experiments is cost. With the recent increases in Illumina per lane read counts (up to 190 million), it is now desirable to pool multiple samples on a single flow cell lane. Based on our previous findings, 30 million reads is sufficient to detect approximately 90% of differentially expressed genes in maize cultivar B73 [11]. For organisms with much smaller genomes such as bacteria and yeast, current read depth/lane is well beyond what is required for accurate gene detection and quantification. Pooling multiple samples into one lane using indices decreases cost and can reduce experimental variation (e.g. lane effects). Additionally, using the methods described in this report and purchasing reagents from alternative sources, it was possible to reduce library construction costs approximately 10-fold from the current manufacture's recommendations (Illumina TruSeq kit). The list of reagents are listed in Table S1. In summary, our improved protocol reduces both the time and cost of library preparation and increases the quantity and quality of reads over standard protocols.

### Comparison to non strand-specific protocols

**Figure 3:**
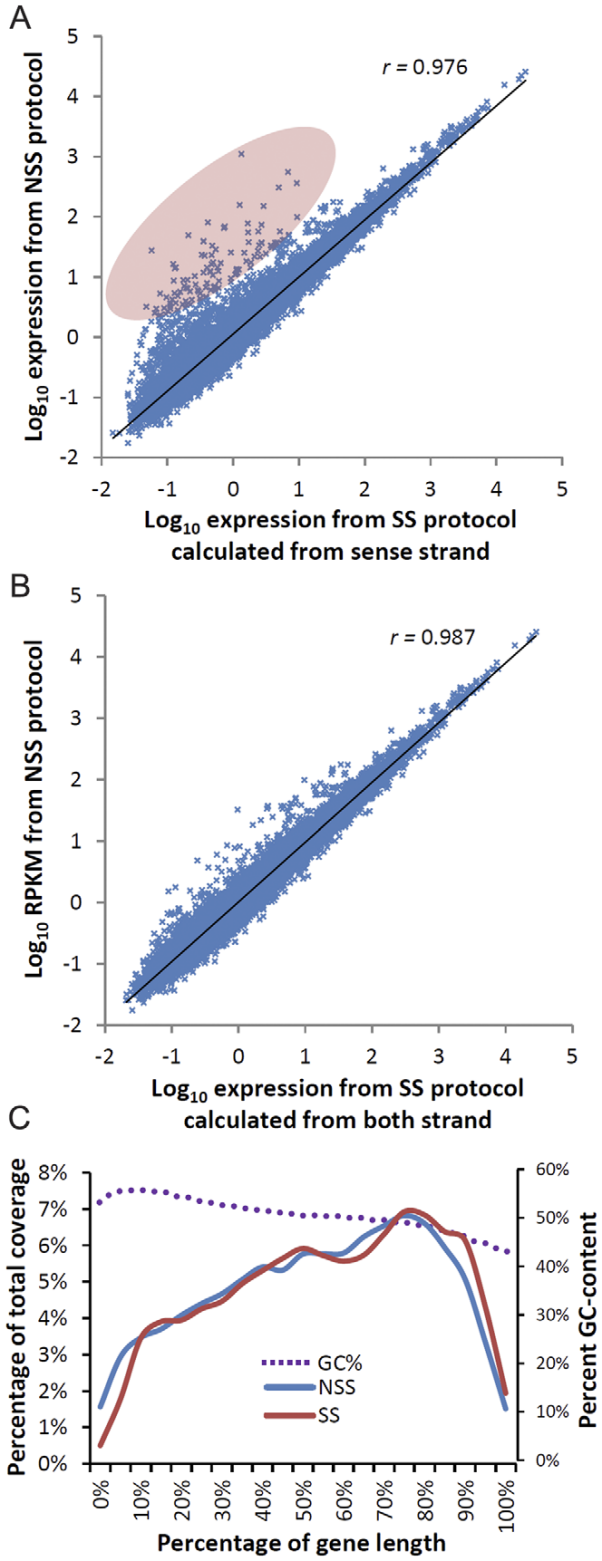
Correlation of RPKM calculated from NSS and SS protocols and coverage statistics. (a) Scatter plot of log_10_ correlation of SS- and NSS-derived RPKM values. SS-derived RPKM values are calculated from the sense strand only. *r* is the correlation coefficient. (b) Scatter plot of log_10_ correlation of SS- and NSS-derived RPKM values. Derived RPKM values are calculated from both strands of SS-data (c) Coverage plot along average gene body from 5′ to 3′ calculated from both NSS and SS methods. The percent GC content is also plotted.

To compare the output from the strand-specific (SS) and standard non strand-specific (NSS) versions, we performed two RNA-seq experiments in parallel using two-week old rice seedling leaf tissue following nearly identical procedures, with the exception of the dUTP labeling step. For the SS protocol, dUTP was used in second strand synthesis, whereas dTTP was used for the NSS protocol. Libraries were sequenced on six lanes of the GAIIx platform (three lanes for each library). Approximately 100 million 35-nt processed reads were generated for each library and RPKM values calculated. As shown in Figure 3A, the correlation of log_10_ RPKM values from the two datasets is high with a correlation coefficient, *r* = 0.976. As illustrated by the shaded portion of the chart in Fig. 3A, there appears to be a subset of genes with higher estimated gene expression values when the NSS protocol was used relative to the SS protocol. We reason that this shift likely occurs when a large number of sense and antisense reads map to the same gene. In the SS protocol only sense alignments are counted, whereas both sense and antisense alignments contribute to the RPKM in the NSS protocol. To test this hypothesis, we re-analyzed the data generated from the SS protocol and added the reads from both sense and antisense strands together. Indeed the new comparison, as shown in the Figure 3B, has a higher correlation with the *r* value of 0.987, suggesting that the NSS method does overestimate RPKM for a subset of genes. To visualize this discrepancy, we examined four genes with high differential RPKM values between NSS and SS protocols. Figure S4 shows alignment profiles using the Integrative Genomics Viewer (IGV; [21]). Interestingly, some of the discrepancies arise from incorrectly annotated gene models. In a few cases, convergent genes were incorporated into the one gene model (Figure S4a–c). In other cases, numerous anti-sense and sense reads mapped to the same gene model (Figure S4d), thus confirming our hypothesis that the SS method is a more accurate method to calculate RPKM values and can be used to validate gene models.

It is important to note that the average gene coverage profile is slightly different for the NSS and SS methods. As shown in Figure 3C, the NSS protocol provides better coverage at the 5′-end compared to the SS-method. This is a consequence of the SS protocol; fragmented RNA molecules are always sequenced from the 3′ end, and depending on the sequence length, sequencing may not proceed to the 5′ end. This bias can be partially negated by generating longer reads (in this study, we used 35-bps reads) or completely overcome with paired-end sequencing, where sequencing starts from both ends. We also observed a higher coverage towards the 3′ end of the average gene body. This is possibly caused by RNA degradation, since we used an oligo-dT based purification method that captures mRNA at the 3′ end. The other possibility is a PCR bias resulting from high GC-content that increases near the 5′ end of rice genes. Indeed, when we plot the average GC content across the average rice gene body, it show a gradual drop from over 50% GC to just over 40% GC (Fig. 3C).

### Analyzing anti-sense alignments

**Figure 4:**
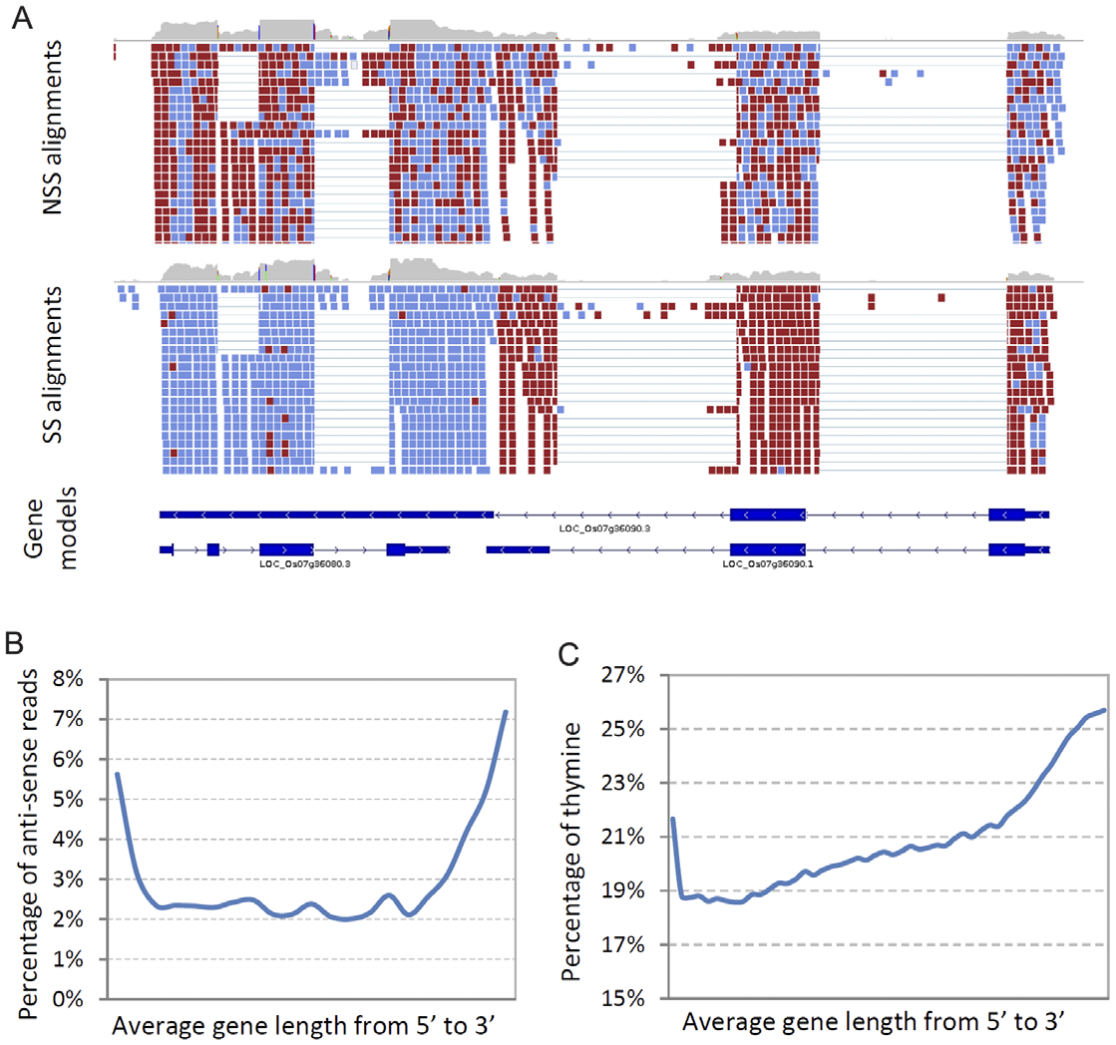
Survey of anti-sense alignments. (a) An example of read alignment showing NSS- and SS-derived data for rice gene Os07g36090. The alignment is visualized using IGV (www.broadinstitute.org/igv/). Red and blue colors designate the directionality of reads. (b) Line plot showing percentage of anti-sense reads aligned to the average rice gene body from 5′ to 3′ end. (c) Line plot showing percent T along average rice gene body from 5′ to 3′ end.

As previously mentioned, we used a slightly modified version of the dUTP method to enforce the strand specificity in our final libraries [20]. We increased the incubation time with UDG (Uracil-DNA Glycosylase) to 30 minutes to enforce the complete degradation of dUTPs. From the 82 million aligned reads that were generated using the SS protocol, we detected approximately 3.88% anti-sense reads according to the most current rice version 6.1 genome annotation [22]. This is slightly higher than the percentage of antisense reads detected in yeast using multiple strand specific protocols [13]. This discrepancy may reflect a true biological difference or a technical limitation related to the maturity of the genome annotation. That is, annotation for the yeast genome is highly refined, enabling a very accurate mapping of antisense reads to the gene space. Given the fact that the rice genome annotation is still being improved, some of the anti-sense reads are due to incorrectly annotated gene models. Figure 4A shows an example where an incorrectly annotated gene model contributes to the over-estimation of anti-sense coverage. Based on sense-strand alignments, the upper gene model is likely incorrect (Os07g36090.3). The other two gene models (Os07g36080.1, Os07g36090.1) running opposite directions are supported by the aligned reads. In this case, if Os07g36090.3 is used for calculating the anti-sense alignment, a substantial number of reads would align to the opposite strand. This example clearly demonstrates the advantage of utilizing a strand-specific protocol, as it is difficult to resolve the validity of gene models with only NSS reads alignment (compare top and middle panels in Fig. 4A).

The other interesting aspect of anti-sense alignments as detected by the SS-protocol is the distribution of reads relative to the transcript model. As shown in Figure 4B, a large number of anti-sense reads map to transcript termini (likely the UTR regions). We considered the possibility that a lower thymine (T) content at the ends of genes may have resulted in this profile, as regions with few Ts would serve as poor substrates for the UDG. However, this is clearly not the case, as the average percentage of T is in fact higher at transcript ends (Fig. 4C). Previous reports in animals and yeast also support our observation that antisense transcripts are more abundant in the 5′ and 3′ UTR regions, and these anti-sense transcripts likely play regulatory roles in modulating gene expression [23], [24], [25]. Although similar analyses have not been published for plant species, our results suggest that comparable mechanisms are employed in rice.

The validity of anti-sense transcripts captured by strand-specific RNA-seq protocols is of great interest to the research community. To examine this within our data, we first compared the average percentage of anti-sense transcripts captured relative to the total transcript pool as shown by Levin *et al* [13]. Our analysis shows a relatively constant 2% anti-sense transcripts over the middle potion of rice genes, which is comparable to previous findings in other species [13]. Yet, we cannot rule out the possibility that these detected anti-sense reads are a baseline of anti-sense “noise” generated from either the limitations of the technique or a low level of antisense transcription present throughout the genome [26], [27], [28]. Interestingly, we also detected 1.88% of anti-sense alignments from the two most abundant aRNA spike-ins synthesized by *in vitro* transcription, which technically should not generate anti-sense reads. While it is possible that this is an artifact of our experimental method (e.g. incomplete digestion), it is still likely that the synthesized aRNA would contain a finite amount of anti-sense transcript (e.g. through template switching).

It is important to note that our results are derived from oligo-dT enriched mRNA populations, and it has been reported that most natural antisense RNA are not polyadenylated in mouse [29] and possibly in other organisms, suggesting there may be more anti-sense transcripts that escape detection using our approach. Alternative non oligo-dT-based mRNA enrichment methods (e.g. rRNA depletion) would overcome this limitation and provide a more complete coverage of natural anti-sense RNA.

### Multiplexing using indexed adapters

**Figure 5:**
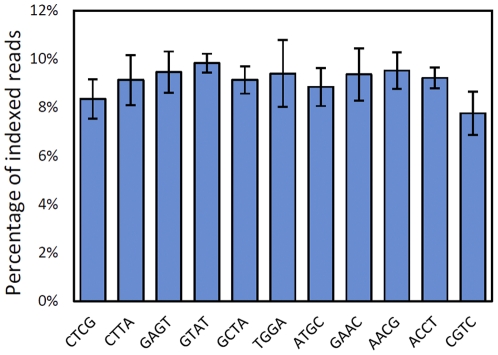
Average multiplex read distribution. Bar plot of the read distribution among the eleven indices used for this study. Y axis represents the average percentage of indexed reads relative to the total number of reads from each lane. X axis shows the index sequences. Data is averaged from six lanes of data with standard error shown.

One of the challenges of multiplexing samples is ensuring an even distribution of read counts across indexed libraries [30]. In the method described here, a combination of five nucleotides serves as the index with a T as the common fifth base pair to minimize biases caused by differences in ligation efficiency. The index itself is incorporated into the adaptor as illustrated in Figure S5. When samples are multiplexed using this design, they can be processed using single-end or pair-end sequencing on the Illumina platform. The read output starts with the index and a T followed by the target sequence. We tested a set of adaptors by multiplexing eleven rice leaf samples in one lane and sequenced the libraries using a total of six lanes on a GAIIx Illumina machine. The average ratio of indexed reads are shown in Figure 5. The percentage of reads derived from each of the eleven indices is relatively uniform, which is a notable improvement to an initial study where index adaptors were used (e.g. [31]) and comparable to Illumina's official multiplexing scheme with a lower cost. It is worth noting that the edit distances, or number of changes to transform one index sequence into another, are at least two among the 11 indices. Thus, with one sequencing error in the first five bps, a read will be assigned to a unique index. This is important, as we have observed higher errors rates at the 5′ and 3′ ends of reads. By including reads with one mismatch to the index, it was possible to reclaim an additional 5% of the total mappable reads (4.8 million reads).

One drawback of our current index design is that it is incompatible with the new calibration method used on the Hiseq2000 platform. Instead of using the first base for calibration as in the GAIIx platform, HiSeq2000 uses the first five bases. Since the fifth base of the multiplexing index is always T, the calibration software overcompensates for the bias and no longer accurately calls subsequent reads. We have explored two approaches to resolve the compatibility issues. The most straightforward is to adapt a longer index design. Using this method we have tested four balanced 7-bps indices that performed well on the HiSeq2000 machine. From a single lane of HiSeq2000 sequencing, we captured 72,903,160 reads, of which 71,960,164 could be unambiguously associated with a specific index (98.7% of total reads) (Figure S6). Another way to overcome the calibration issue is to spike approximately 5% PhiX control (a common control sample for Illumina platform) into each lane with the 5 bps indices. Although we have used a PhiX spike-in successfully (Pinghua Li, personal communication), it nevertheless leads to a loss of total reads. Thus, with slight modifications our indexing design can be applied to the HiSeq2000 platform.

Illumina's official multiplexing protocol and kit enable one to pool of up to twelve samples, and the output can be deconvoluted to individual samples. While Illumina's approach to multiplexing is adequate, the associated costs of using official Illumina library construction kit become inhibitory for many labs when a large number (e.g. hundreds) of libraries are constructed.

### Detecting significantly expressed genes

**Figure 6:**
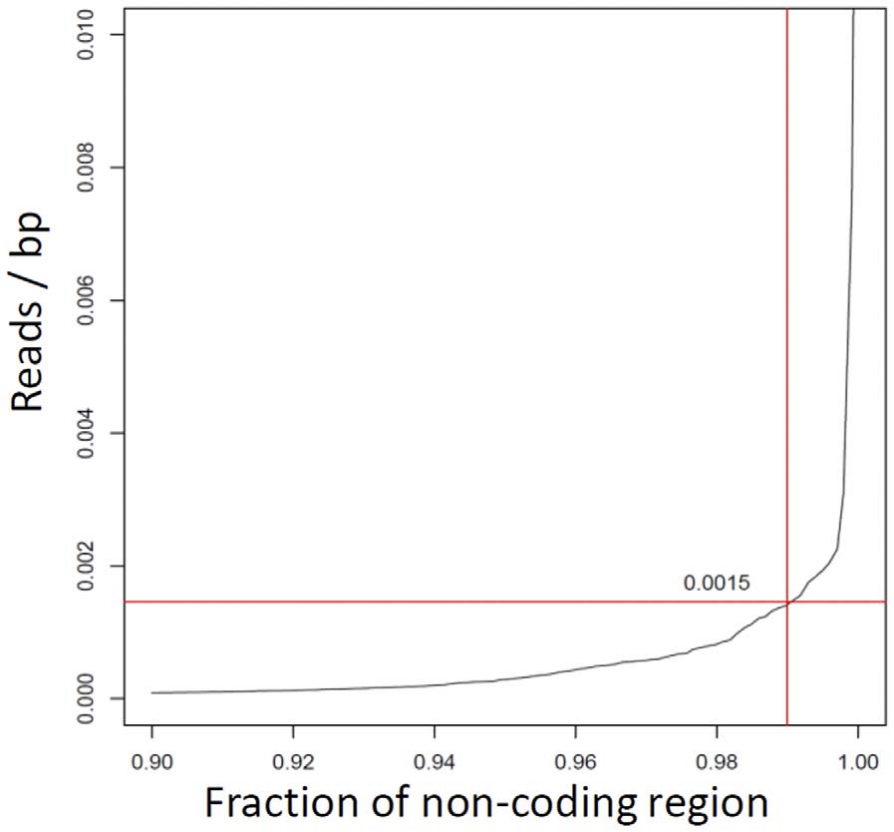
Determination of the background cutoff for RNA-seq data. Y axis shows the number of reads that map to non-coding regions relative to the total bp of non-coding regions (NCR). The x-axis displays the fraction of NCRs. As shown, 99% of the regions denoted as non-coding have fewer than 0.0015 reads/bp.

An emerging need for RNA-seq data analysis is to determine confidence intervals for defining significance in gene expression values. As a part of our data analysis pipeline, we determined the significantly expressed genes by comparing the reads that aligned to the annotated gene space (i.e. exons and UTRs) to the “non-coding regions” (NCRs) that are defined as regions of at least 5 kb away from any annotated genes. Our method is built upon two assumptions: first, the NCRs, by definition, do not generate a large number of transcripts; second, that gene annotation is accurate. Based on these assumptions, the reads mapped to the NCRs are likely due to artifacts of the sequencing method or library construction. For instance, DNA contamination in the RNA samples could lead to read placements in NCRs and thus can be used to estimate the background or “noise” level. As shown in Figure 6, we calculated the significance of gene expression using the normalized coverage in 99% of the NCRs (see methods for more detail). Using an empirical Bayesian method based on a Poisson distribution of reads, we calculated the posterior odds (B) for all genes and consider a gene as expressed if the B value is less than 1 (see methods section and Figure S8 for detail).

**Figure 7:**
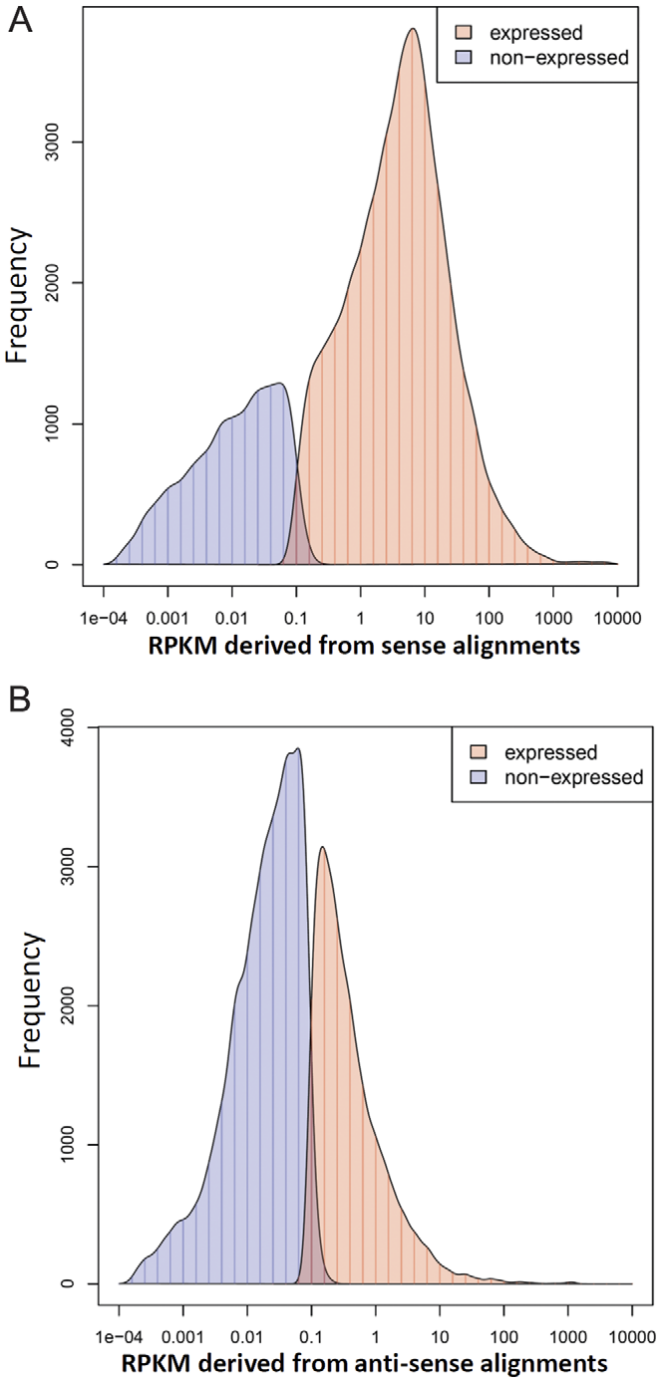
Distribution of reads mapping to expressed and non-expressed genes. (a) Distribution of sense alignments and (b) anti-sense alignments. The RPKM values were calculated from three replicates. The frequency shows the number of gene models per bin (vertical bars). A smoothed curve is plotted. Genes with average RPKM equal to zero are not shown in the histograms.

For SS-derived data, we detected 34,455 sense transcripts and the corresponding false discovery rate (FDR) was estimated as 0.6%. The distribution of estimated gene expression values are shown in Figure 7A and indicates that an RPKM value of approximately 0.3 or greater is sufficient for defining a gene as “expressed”. This is reasonable since a value of 0.3 corresponds to approximately 32 reads aligning to an average 1.1 kb gene model from a combination of approximately 90 million mapped reads.

Using the same approach, we identified 14,704 significantly expressed anti-sense transcripts (FDR = 5.6%). The average expression levels of detected anti-sense transcripts are much lower than that of the sense transcripts (Fig. 7). All significantly expressed rice genes and anti-sense transcripts are listed in Table S2 and S3. Additionally, we inspected a few significantly expressed anti-sense transcript using IGV and further verified them by RT-PCR as shown in Figure S7.

### Detecting Intronic transcription

Another challenge in RNA-seq data analysis is interpreting reads that map to intronic regions. Intronic reads are likely of biological importance given the frequency of observed intron retention events [11], [32]. For instance, it is known that approximately 42% of intron-containing genes in *Arabidopsis* and maize are alternatively spliced. A subset of these isoforms appear to be under developmental control or may be regulated by abiotic stress [32].

In order to survey the intronic alignments, we constructed a database of intronic sequences from unique gene models and performed an alignment with the SS-reads. From a total of 97,362,750 aligned reads, 7,373,258 (approximately 7.57%) mapped to intronic regions. Reads that map to introns may indicate alternative splicing and pre-mRNA populations but may also indicate incorrectly annotated gene models. To assign a significance value to a given intron, we developed a novel statistical model and an empirical Bayes method, similar to the one used to detect significantly expressed genes, to detect significant intronic alignments. For this test, we considered a number of factors that would affect intron detection that include: 1) RPKM value of the corresponding gene 2) intron length and 3) and number of reads mapped to the intron. As described in the Methods section, we detected 27,182 expressed introns (approximately 12.8% of all introns) from the SS-method derived RNA-seq data with an estimated FDR level of 4.8% (Figure S9; Table S4). We also visualized a few significantly expressed introns using the IGV and verified the presence of introns in mature transcripts with RT-PCR as shown in Figure S7.

### Computation pipeline for RNA-seq data analysis

**Figure 8:**
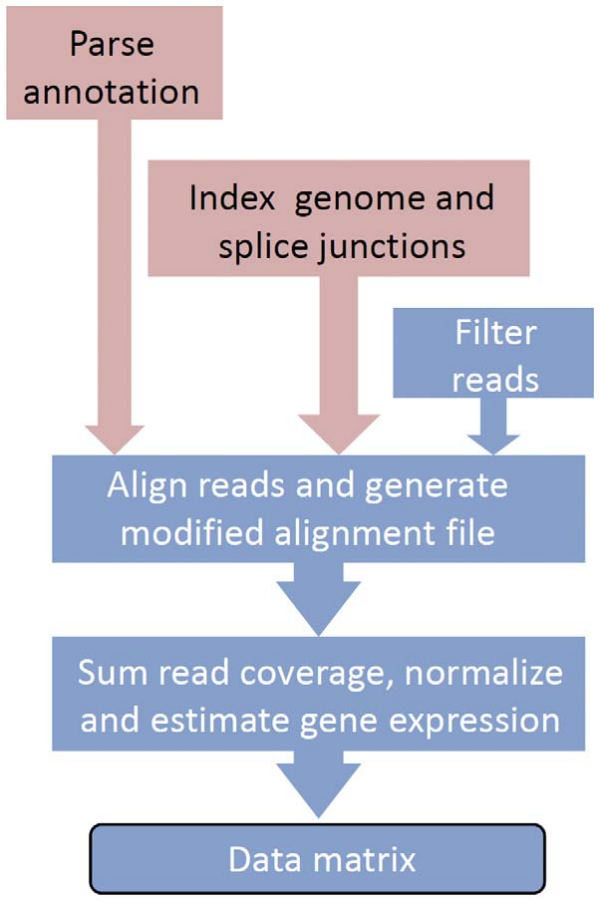
Data analysis pipeline. Pink boxes highlight functions that are based on genome sequence and annotation and are not dependent on experimental data. Blue boxes highlight functions executed for each experimental run.

We have compiled our data analysis pipelines into an integrated package of annotated Perl and R-based modules. The source codes are easily accessible and adaptable from (http://c3c4.tc.cornell.edu/resource.aspx). It is also possible to run the script directly from iPlant website (Matt Vaughn, personal communication), providing a mechanism for community access. The overall flow of the pipeline is illustrated in Figure 8. Importantly, these scripts are well documented and can be easily modified and improved as RNA-seq technologies advance. As such, we have intentionally left the pipeline highly annotated and modular, so that modification and improvement can be easily made. Thus, this computational pipeline can serve as a useful resource that the community can improve and adapt upon and may accelerate the unification of RNA-seq data analysis.

## Methods

### Protocol for the library construction

The complete protocol for multiplex Illumina sequencing-read library construction is compiled in a PDF available for download as File S1. The reagents used in this protocol are also listed in Table S1.

### Rice growth and harvesting

*Oryza sativa* Nipponbare (rice) seeds were used for expression profiling. Husks were first removed from 5 to 10 g of seeds using a palm de-husker and extracted seeds were soaked in 75% ethanol for 1 minute at room temperature and then soaked in 3% bleach (sodium hypochlorite) solution at 30°C for 30 minutes. The seeds were then washed 6 times with tap water before adding 30 mL of tap water and soaking at 30°C overnight with mild agitation. The following day the tap water was changed every 3–4 hours and again left overnight for incubation at 30°C. On the third day, the tap water was changed twice and approximately 3 hours after the last change of water, the seeds were planted in flats of water-soaked soil consisting of 1 part Unimix (growing media nutrient charge), 5 parts OsmocotePlus (15-9-12 fertilizer), 5 parts lime, 9 parts quartz sand, 9 parts top soil, 26 parts Turface (75% montmorillonite clay), 443 parts peat moss and 933 parts vermiculite. The seeds were planted 3 cm apart with the embryo orientated downward. The plants were grown in a BDW-40 chamber (Conviron, Manitoba, Canada) under an 80∶20 mix of metal halide and 100w capselite halogen lamps at a light intensity of 550 µmol/m^2^/sec. The temperature in the light was 31°C, the temperature in the dark was 22°C, and the relative humidity was 50%. The plants were watered as needed and grown for 14 days with a 12-hour day/night cycle.

Tissue samples were collected 2 hours after the daylight cycle had begun. The third leaf was excised from the plants with a razor blade, cut into 2 cm sections and immediately placed into liquid nitrogen submerged tubes. Three biological replicates were collected and each replicate contained tissue from approximately 40 rice seedlings grown over a separate 14-day period.

### RNA isolation

Eight metal ball bearings were added to each tube (SARSTEDT, catalog number 60-540-016) that contained rice leaf tissue and capped tubes were placed into an Nalgene 1000 mL plastic Jar (Sigma, catalog number Z380334) and plunged into liquid nitrogen. Several holes were drilled into the side of the Nalgene Jar to allow release of vaporized liquid nitrogen. They were then placed into a Harbil 5G-HD paint shaker (Fluid Management, IL USA) and shaken vigorously 9 times for three minutes intervals. Liquid nitrogen was replenished during the third and sixth mixes to keep the tissue at low temperature. At the end of the shaking, the homogenized rice tissue and tubes were transferred onto dry ice for further processing.

Trizol (6.5 mL) was added to each tube containing plant tissue on dry ice (7.5 ml of Trizol for 1 g of tissue). Tubes were vortexed at the highest speed until the Trizol and plant tissue were completely homogenized in a liquid state, and then placed at room temperature for 10 minutes. HPLC-grade chloroform (1.3 mL) was added to each tube and inverted to mix. The tubes were placed at room temperature for another 5 minutes and then centrifuged at 5000×g for 30 minutes at 4°C using a Hermle Z383 centrifuge. After centrifugation, the upper aqueous phase was transferred to a new tube, an equal volume of HPLC-grade isopropanol (roughly 3.5 mL) added, and tubes left on ice for 30 minutes. The mix was then centrifuged again at 4°C at 14,000× g in a Sorvall RC5C plus centrifuge with appropriate adapters. After removing the supernatant, the precipitated pellets were washed twice with ice-cold 75% ultra-pure and RNAse free ethanol. In between washes, the pellets were centrifuged at 5,000× g for 10 minutes. The pellets were re-suspended in 0.05–0.3 mL RNA-secure™ solution (Ambion, CA) based on the pellet size and heated at 60°C for 10 minutes before stored at −80°C in a freezer.

### Purification and fragmentation of mRNA

The starting total RNA concentration was measured using NanoDrop (Thermal Scientific, DE) and 15 µg of total RNA was used for mRNA purification (an optional DNAse-treatment using the on-column DNAse treatment provided by Qiagen did not show statistically detectable differences in final products). The total RNA was topped with nuclease-free dH_2_O to 50 µL and heated at 65°C for 2 minutes and immediately chilled on ice. 30 µL of Dynabeads (Invitrogen, CA) were washed twice with 100 µL of binding buffer (Tris-HCl 20 mM, LiCl 1 M, EDTA 2 mM) and re-suspended in 50 µL of binding buffer. The RNA samples were then mixed with the Dynabeads mixture and incubated at room temperature with mild agitation for 10 minutes. Beads that bound mRNA were separated from the supernatant using a magnetic stand (Invitrogen, CA). The beads were then washed twice with 150 µL of washing buffer (Tris-HCl 10 mM, LiCl 0.15 M, EDTA 2 mM), and eluted with 50 µL elution buffer (Tris-HCl 10 mM) at 80°C on an incubator/shaker for 2 min with mild agitation. The elution was treated again as starting RNA material and the above procedures were performance one more time to ensure mRNA purity. mRNA samples were then eluted in 16 µL of elution buffer. The concentration of mRNA was immediately measured with 1 µL of sample in a Qubit® Fluorometer (Invitrogen, CA). 30 ng of mRNA was used for cDNA synthesis and the remaining mRNA samples were immediately stored in −80°C freezer. The volume of mRNA samples were adjusted to 4 µL. Four µL of spiking aRNA mix and 4 µL of 5× first strand buffer (Invitrogen, CA) were then added to each tube. The mixture was incubated at 94°C for 5 minutes to fragment RNA and then immediately chilled on ice before the next step.

### Synthesis of dUTP-marked dsDNA

The 12 µL of fragmented mRNA, 0.5 µL of Random primer (Invitrogen, CA), 0.75 µL of SupeRase-In (Ambion, CA) and 1 µL of DTT (100 mM) were heated at 65°C for three minutes in a PCR machine. At the end of incubation, 4 µL of water, 1 µL of DTT (100 mM), 0.1 µL of dNTPs (25 mM), 0.5 µL of SupeRAse-In and 0.5 µL of Superscript II (Invitrogen, CA) were added and incubated in a PCR machine using the following conditions: 25°C for 10 minutes, 42°C for 50 minutes, 70°C for 15 minutes and a 4°C hold. The product was then purified with RNAClean XP beads (see section **SPRI bead-based purification and size-selection** for details) and eluted with 16 µL nuclease-free water. The RNA/cDNA double-stranded hybrid was then added to 2 µL of 10× NEB buffer-2 (NEB, MA), 1 µL of dUTP mix (10 mM dATP, dCTP, dGTP and 20 mM dUTP), 0.5 µL of RNAse H (2 U/µL), 1 µL of DNA polymerase I and 0.5 µL of DTT (100 mM). The mixture was incubated at 16°C for 2.5 hours. The resulting dUTP-marked dsDNA was purified using 38 µL of AMPure XP beads and eluted with 32 µL EB buffer (10 mM Tris-Cl, pH 8.5) and saved in the −80°C freezer until the next step.

### End repair, dA-tailing and adaptor ligation

The purified dsDNA (16 µL) was mixed with 2 µL of 10× End Repair Buffer (Enzymatics, MA), 1 µL of dNTP mix (10 mM each) and 1 µL of End Repair enzyme mix (Enzymatics, MA). The mixture was incubated in a PCR machine for 30 minutes at 20°C and purified with 28 µL of AMPure XP beads and eluted with 17 µL of nuclease-free water. It was then added to 2 µL of 10× NEB buffer-2 (NEB, MA), 1 µL of 10 mM dATP mix, and 0.5 µL of Klenow 3′–5′ exo^−^ (Enzymatics, MA). The mixture was incubated in a PCR machine at 37°C for 30 minutes, then purified with 28 µL of AMPure XP beads and eluted with 10 µL of nuclease-free water. The 10 µL of end-repaired and dA-tailed product was then added to a mixture of 1 µL of indexed adaptor (See **making indexed adapter** section and Table S5 for detail), 12 µL of 2× ligation buffer and 1 µL of T4 DNA ligase (Enzymatics, MA). The final mix was incubated at 20°C for 20 minutes in a PCR machine. Half of the product was saved in the −80°C freezer as backup. The other half (12 µL) was mixed with 12 µL of “12p XP” beads (see section **SPRI bead-based purification and size-selection** for details) and incubated at RT for 6 minutes. The supernatant was then mixed with 12 µL of AMPure XP beads and 5 µL of 40% of PEG8000 and eluted with 10 µL of nuclease-free water, it was then again purified using 12 µL of AMPure XP beads and eluted in 30 µL of EB buffer. Half of the product (15 µL) was saved in the −80°C freezer as backup.

### dUTP excision and amplification of library

The size-selected dsDNA product (15 µL) was mixed with 1 µL of uracil DNA glycosylase (Enzymatics, MA) and incubated at 37°C for 30 minutes in a PCR machine. Without purification, the mixture was then added to 2 µL of Illumina PE primers (5 µM each) (Table S5), 6 µL of 5× Phusion HF buffer, 1 µL of 10 mM dNTP, 1 µL of Phusion® Hot Start 2 High-Fidelity DNA Polymerase (NEB, MA) and 4.5 µL of water. The PCR mix was incubated with a programmed cycle as following: 94°C for 30 sec, 11 cycles of 98°C, for 10 sec, 65°C for 30 sec, 72°C for 30 sec; 72°C for 5 min followed by a hold at 4°C. The final product was purified with 43 µL of AMPure XP beads and eluted with 12 µL of EB buffer.

### Mixing library with different indices

The concentration of PCR products was measured using the dsDNA-HS protocol on the Qubit Fluorometer. Equal quantities of libraries (approximately 5 ng per sample) with different indices were mixed and stored in −80°C freezer before sequencing.

### SPRI bead-based purification and size-selection

SPRI beads used for this experiment were purchased from Beckman Coulter (CA, USA). RNAClean XP was used for the cleaning of the RNA/DNA hybrid product before second strand synthesis and AMPure XP was used for all other purification steps. For purification, with the specified amount of SPRI-beads added to each purification, 50% (v/v) of pure ethanol was added to the mixture. The mixture was vortexed and kept at room temperature for at least 5 minutes before being placed on a magnetic stand to separate the SPRI-beads from the supernatants. Once the supernatant was removed, the beads were washed twice with 100 µL 75% ethanol and quickly dried with gentle airflow above the tubes and subsequently re-suspended with water. The elution suspension was incubated for 2 minutes at RT and then placed on the magnetic stand, the supernatant was removed and placed in a new tube.

For size-selection using the SPRI beads, we replaced the stock buffer of AMPure XP beads with a 12% PEG-8000 and 2.5 M NaCl solution (“12p XP” buffer). One mL of Ampure XP beads were placed on a magnetic stand and left for 10 minutes. The supernatant was then removed and the beads were washed twice with ultra-pure water and re-suspended in the “12p XP” solution. When “12p XP” beads were used, the beads were discarded, while the supernatant is retained for subsequent applications.

### Making indexed adapters

DNA Nucleotides were ordered from IDT with specific modifications (see Table S5 for details). Hybridization of indexed adapters was performed in hybridization buffer (0.1 M NaCl, 10 mM Tris-HCl, 10 mM EDTA, pH 8,0) with a thermal cycler. The hybridization program was as following: 75°C for 5 minutes, ramping down to 25°C with 1°C per second for 50 minutes, and holding at 25°C for 30 minutes. They were frozen at −20°C before use.

### RT-PCR verification of anti-sense transcript and intronic alignments

Total RNA was extracted from rice seedling leaf tissue as described above. First stand cDNA synthesis was performed using gene-specific primers as listed in Table S5. Total RNA was treated with Turbo DNase (Ambion, CA) following manufacturer's recommendations and 200 ng was then used for subsequent first-stand cDNA synthesis. Briefly, 4 µL of total RNA was mixed with 1 µM of gene specific primers, 1 µL of 10 mM dNTPs, 4 µL of water and 1 µL of RNAse-OUT (Invitrogen, CA) and incubated at 65°C for 5 minutes and immediately chilled on ice. The reaction mix was then added to 4 µL of 5× first strand buffer (Invitrogen, CA), 2 µL of 0.1 M DTT, 1 µL of RNase-OUT and 1 µL of SuperScript III (Invitrogen, CA). The reaction mix was then incubated at 50°C for 50 minutes, 85°C for 5 minutes followed by 4°C hold in a thermal cycler. The first-strand cDNA product was incubated with RNase-H (Invitrogen, CA) at 37°C for 20 minutes and used as the temple for PCR. Each PCR reaction was performed using 4 µL of first-strand cDNA template along with 11 µL of water, 10 µL of primer (2 µM each) and 25 µL of 2× GoTaq mix (Promega, WI). PCR cycles were optimized for individual genes ranging from 25 to 32 cycles.

### Determining significantly expressed genes

Let 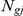 denote the observed number of reads for gene *g*, and technical replicate *j* of a sample, where *g = 1, 2, 3, …, G*, *G* is the total number of genes that are supported by mapped reads and *j = 1,2,..,n, n* is the number of replicates. Following previous reports, we assume 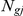 follows a Poisson distribution [33], [34]. By properties of Poisson distribution and independence between replicates, 
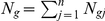
 also follows a Poisson distribution. To detect the expressed genes based on the background expression, we model the mean of the Poisson distribution for 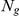 as 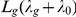 where 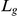 is the length of gene *g*, 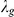 is the true expression level of gene *g*, and 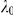 is the background expression level. For each sample, we examine whether gene *g* is expressed by testing 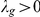 versus 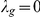.

The number of genes, *G*, is much higher than the number of replicates *n* for each sample. To improve the performance of the test, we used an empirical Bayes approach that uses information obtained from expressed genes to inform the analysis. Similar ideas were used to test for differentially expressed genes in microarray data analysis [35], [36]. Let 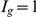 where gene *g* is expressed and supported by RNA-seq data and 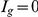 if the gene is not expressed but may have mapped reads that arise from experimental artifacts. Hence, 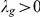 corresponds to 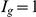 and 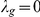 is equivalent to 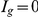. Furthermore, we assume that 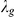 of expressed genes follow a Gamma distribution, i.e.,
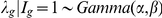
where 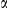 and 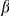 are parameters for the Gamma distribution with mean 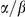. Hwang and Liu showed that the maximum average powerful (MAP) test in such multiple testing scenarios can be approximated by the empirical Bayes likelihood ratio test [36]. A monotonic transformation of the test statistic under our model gives the posterior odds 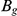:
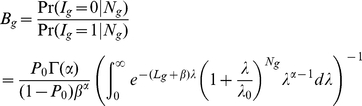
where 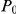 is the proportion of non-expressed genes. We report the genes with 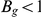 as detected. The associated FDR level is estimated using the posterior probabilities [37]. The posterior odds and associated FDR for sense and anti-sense transcripts are plotted in Figure S8.

To calculate 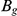, we first estimated the values of the parameters 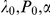 and 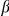. The background expression level, 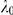, was estimated based on the genomic regions that are at least 5 kb away from any annotated exon and we call such regions non-coding regions (NCR). We define the length-normalized coverage (LNC) for a genomic region as the ratio of total number of mapped reads to the length of the region. The plot of the distribution of LNC for the NCRs in Figure 6 shows a dramatic increase above the 99-percentile. This is likely due to incorrectly annotated gene models where reads are mapped. Therefore, we excluded such regions and obtained the ratio of the total mapped reads and the total length for the remaining NCRs. The parameter 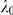 was estimated to be this ratio.

The parameter 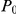 was calculated by the proportion of genes with LNC lower than the estimated background expression level, 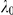. Given the estimate of 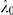, we obtained the maximum likelihood estimate for 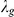 using the genes with higher LNC and these estimates were subsequently used to calculate the maximum likelihood estimates for 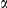 and 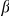 of the Gamma distribution.

### Determining intron retention events

We proposed a novel statistical model to detect intron retention events and derived an empirical Bayes test based on the model. Let 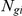 denote the sum of the observed number of reads across replicates for a sample for the *i*-th intron of gene *g* where *i = 1, 2, …, n_g_* and *n_g_* is the number of introns for gene *g*. Assume 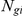 follows a Poisson distribution with mean 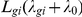 where 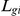 is the length of this intron, 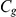 is the expression value of the gene and we used the sum of RPKM values across replicates to approximate it, 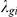 is the rate of the intron retention and 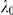 is the *background* level. We normalized the intron retention rate by gene expression under the assumption that intronic alignment is proportional to the gene expression level. Using empirical Bayes approach with a Gamma(a,b) prior for 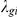,we derived the following posterior odds to test whether an intron retention is detected or not:
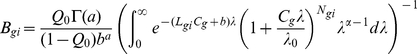
where 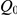 is the proportion of non-expressed introns. The parameters 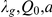 and 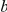 were estimated in the same fashion as described in the previous method section. Introns with 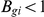 were reported as having significant intron retention and the associated FDR level was estimated as in Gadbury *et al* [37] using the posterior probabilities. The posterior odds and associated FDR for intronic reads are plotted in Figure S9.

## Supporting Information

Figure S1 **Comparison of normalization methods for estimating gene expression.** The comparison is performed using goodness-of-fit statistics using the SS-derived RNA-seq data. Gene expression is normalized using TMM, Q3, Total Reads or Spike-in. The x-axis shows the quantiles of the statistics predicted by the Chi-Square distribution, and the y-axis shows the observed quantiles calculated from RNA-seq data. Perfect match indicates the theoretical scenario when no differential expression is detected among the three technical replicates.
(TIF)

Figure S2 **RNA fragmentation using 1^st^ strand cDNA buffer.** The graphs show bioanalyzer results of fragmented mRNA size distribution following 3, 5 and 10 minutes of incubation. Blue line indicates 200 bps.
(TIF)

Figure S3 **Library size selection using modified SPRI buffer.** Top shows the EtBr-stained gel image of final library sizes using different concentrations of PEG-8000 in the modified buffer. Lane 1 is the control without any input DNA. Lanes 2–6 show results of using 8%–12% PEG-8000 in the buffer. Bottom Image shows ImageJ analysis result of the intensity distribution of the gel image.
(TIF)

Figure S4 **Examples of read alignments from NSS and SS RNA-seq methods.** The four images display alignments visualized using IGV for rice gene model (a) Os01g37920.1, (b) Os01g68490.1, (c) Os03g01020.1 and (d) Os04g55150.2. Each panel shows the genomic region, NSS read alignment, SS read alignments, and gene models. Red and blue colors designate the directionality of reads.
(TIF)

Figure S5 **Schematic of multiplexing adaptor.** Green and blue nucleotides represent the non-complementary arms. Black shows the paired region, and red represents the index sequences. Purple T is the non-paired overhang at the 3′-end and p stands for phosphorylation at 5′-end.
(TIF)

Figure S6 **Read distribution from 7-bp indices on the HiSeq2000 platform.** Each bar represents the absolute number of deconvoluted reads from one lane of HiSeq200.
(TIF)

Figure S7 **IGV visualization and RT-PCR verification of significantly expressed anti-sense transcripts and introns.** (A–D) Screenshots of IGV showing (A) Os0501600.1 (Actin), (B) Os01g48220.1, (C) Os012g18729.1 and (D) Os012g19381.1. Red arrows indicate the primer pairs used to detect intronic expression. Blue arrows indicate the primers used for directional exonic expression detection, and the dashed primers were also used as the gene-specific primer for antisense first-strand cDNA synthesis. The black arrow indicates the gene-specific primer for sense first-strand cDNA synthesis. (E) Gel images of the RT-PCR results showing the existence of intronic and anti-sense expression. Lanes 1–8 are the results of amplification from Os05g01600.1 as follows: lane 1 sense exon primers(blue) using sense 1st strand cDNA (black); Lane 2 intron primers (red) with sense 1st strand template; lane 3 and 4, – RT negative control of lane 1 and 2; lane 5, exon primer (blue) with anti-sense first-strand cDNA as template (dashed blue primer); Lane 6, intron primers (red) with anti-sense 1st strand template; lane 7 and 8, - RT negative control of lane 5 and 6. Lane 13–20 (Os12g18729.1) and lane 21–28 (Os012g19381.1) follow the exact format of lane 1–8. Lane 9 and 11 shows sense and anti-sense detection of exonic expression of Os01g48220.1, while lane 10 and 12 are the –RT negative controls of lane 9 and 10 respectively. Lane number colored yellow indicate presence of amplified PCR product. DNA ladder of 100, 200 and 300 bps are not labeled with numbers.
(TIF)

Figure S8 **Detection of significantly expressed transcribed genes and anti-sense transcripts.** The plots show the posterior odds distribution, B, and corresponding FDR at each cutoff of the posterior odds for (a) transcribed genes and (b) anti-sense transcripts. At the cutoff values of 1 for posterior odds, the associated FDR levels were estimated to be 0.6% and 3.3% for the transcribed genes and anti-sense transcripts, respectively.
(TIF)

Figure S9 **Detection of significantly expressed introns.** The plot shows the distributions of posterior odds and corresponding FDR at each cutoff of the posterior odds. The cutoff value for posterior odds of B = 1 corresponds to an FDR level of approximately 4.8%.
(TIF)

Table S1 **List of reagents for library construction.**
(XLSX)

Table S2 **List of RPKM, FDR and B values for rice gene models calculated from sense alignment.**
(XLSX)

Table S3 **List of RPKM, FDR and B values for rice gene models calculated from anti-sense alignment.**
(XLSX)

Table S4 **List of RPKM, FDR and B values for detected rice intronic expression.**
(XLSX)

Table S5 **List of nucleotide sequences for multiplex adaptors and RT-PCR primers.**
(XLSX)

File S1 **Protocol for constructing of strand-specific multiplex RNA-seq libraries for Illumina platforms in PDF format.**
(PDF)
