# Correction: A Low-Cost Library Construction Protocol and Data Analysis Pipeline for Illumina-Based Strand-Specific Multiplex RNA-Seq

**DOI:** 10.1371/annotation/e5ef7afc-7e81-4053-8670-1bb3402f63fd

**Published:** 2011-11-17

**Authors:** Lin Wang, Yaqing Si, Lauren K. Dedow, Ying Shao, Peng Liu, Thomas P. Brutnell

The following reference should have been included:

Zhong S, Joung JG, Zheng Y, Chen YR, Liu B, et al. (2011) High-throughput illumina strand-specific RNA sequencing library preparation. Cold Spring Harb Protoc 2011: 940-949.

Both manuscripts describe similar protocols that use a dUTP-incorporation step to generate strand-specific data. Zhong et al. demonstrate the use of their protocol to characterize the tomato transcriptome and in this report the authors detail the use of their method to characterized the rice leaf transcriptome.

The authors would also like to note to the readers that the article below by Craig et al. was duplicated as reference 30 and 31 and should have only been cited once as reference 30:

Craig DW, Pearson JV, Szelinger S, Sekar A, Redman M, et al. (2008) Identification of genetic variants using bar-coded multiplexed sequencing. Nat Methods 5: 887–893.’

