# Supplementary figures and images for: A Low-Cost Library Construction Protocol and Data Analysis Pipeline for Illumina-Based Strand-Specific Multiplex RNA-Seq
Source: PLoS One. 2011 Oct 19;6(10):e26426. doi: 10.1371/journal.pone.0026426 (PMC3198403; doi:10.1371/journal.pone.0026426)

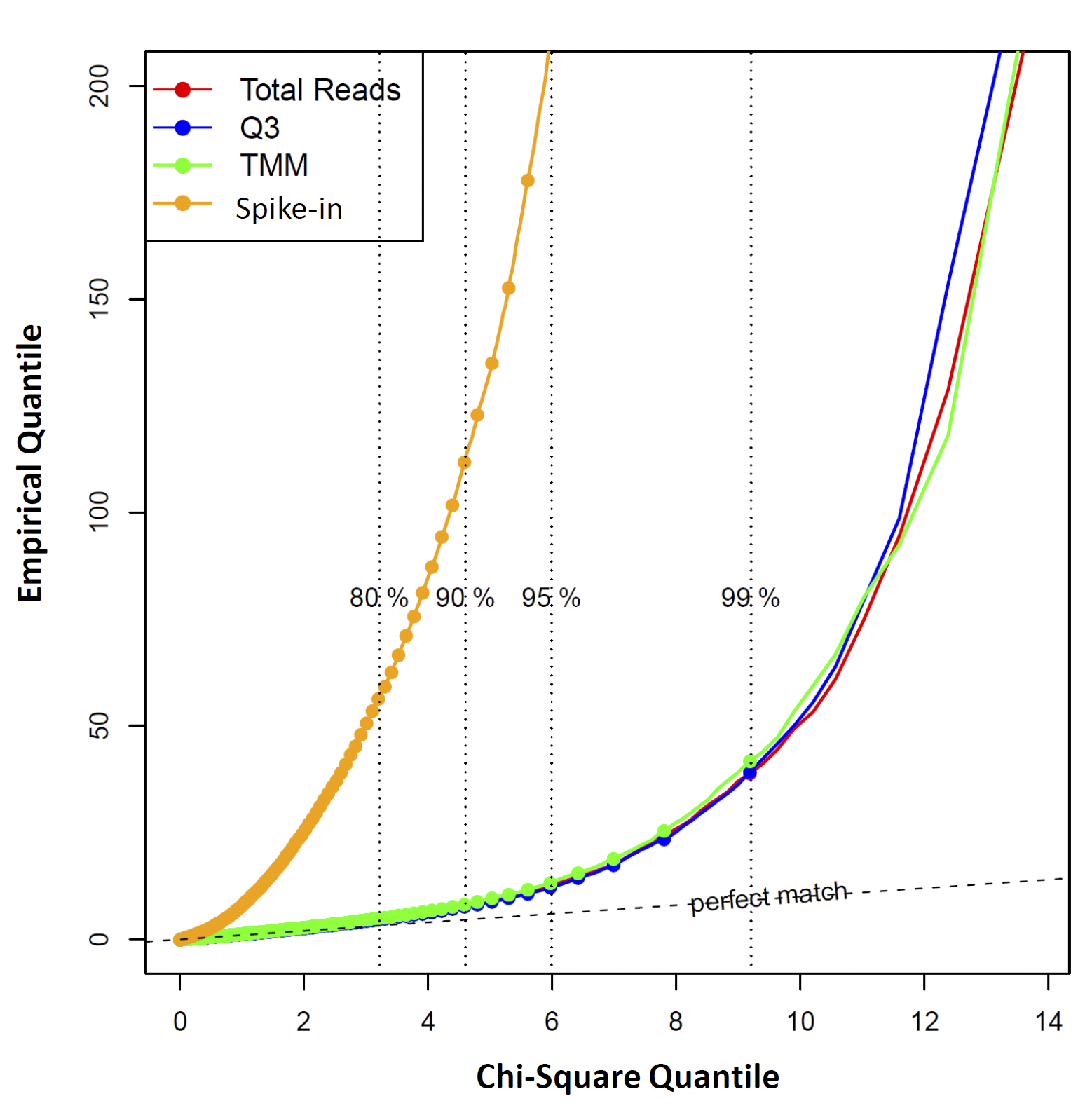

Supplement: Figure S1 — Comparison of normalization methods for estimating gene expression. The comparison is performed using goodness-of-fit statistics using the SS-derived RNA-seq data. Gene expression is normalized using TMM, Q3, Total Reads or Spike-in. The x-axis shows the quantiles of the statistics predicted by the Chi-Square distribution, and the y-axis shows the observed quantiles calculated from RNA-seq data. Perfect match indicates the theoretical scenario when no differential expression is detected among the three technical replicates. (TIF) [file pone.0026426.s001.tif]

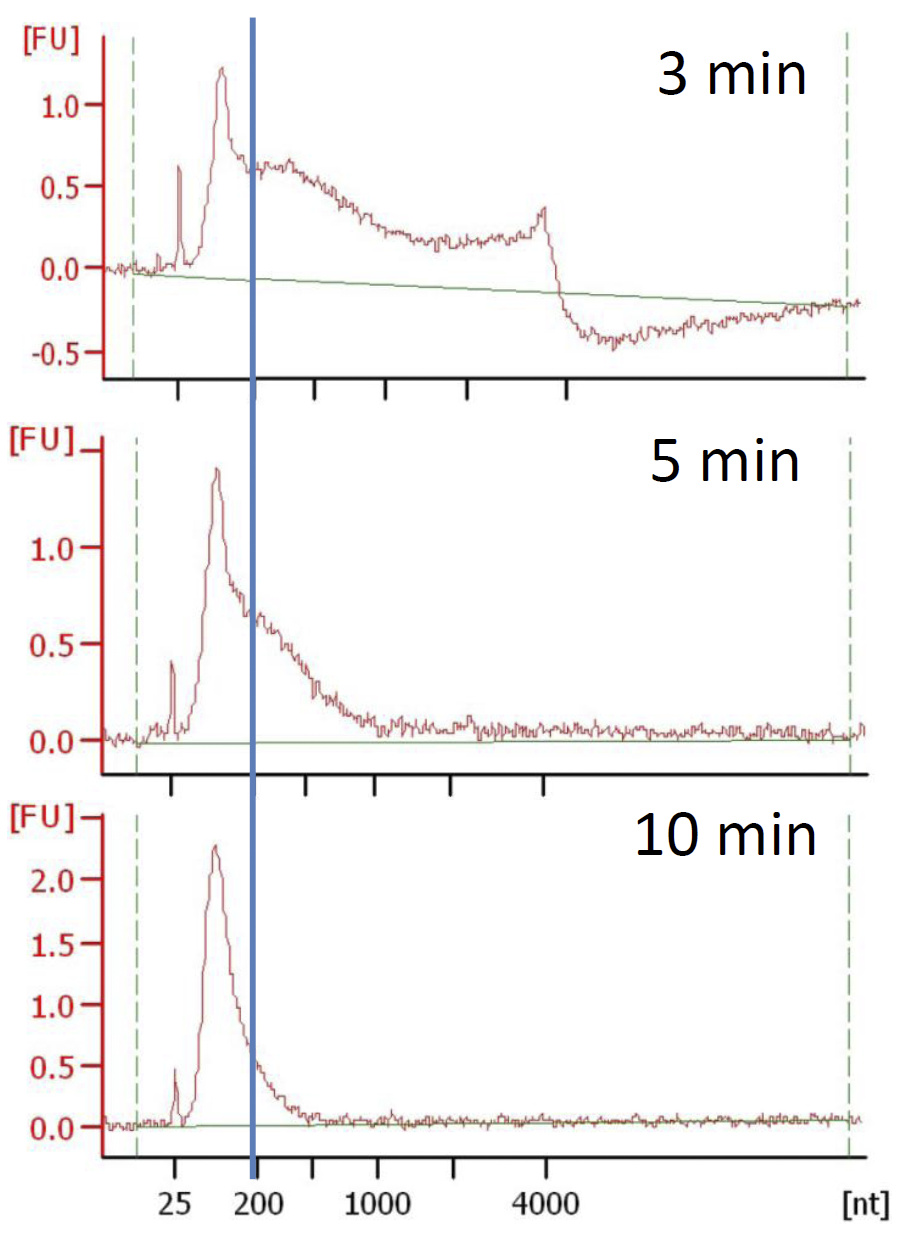

Supplement: Figure S2 — RNA fragmentation using 1st strand cDNA buffer. The graphs show bioanalyzer results of fragmented mRNA size distribution following 3, 5 and 10 minutes of incubation. Blue line indicates 200 bps. (TIF) [file pone.0026426.s002.tif]

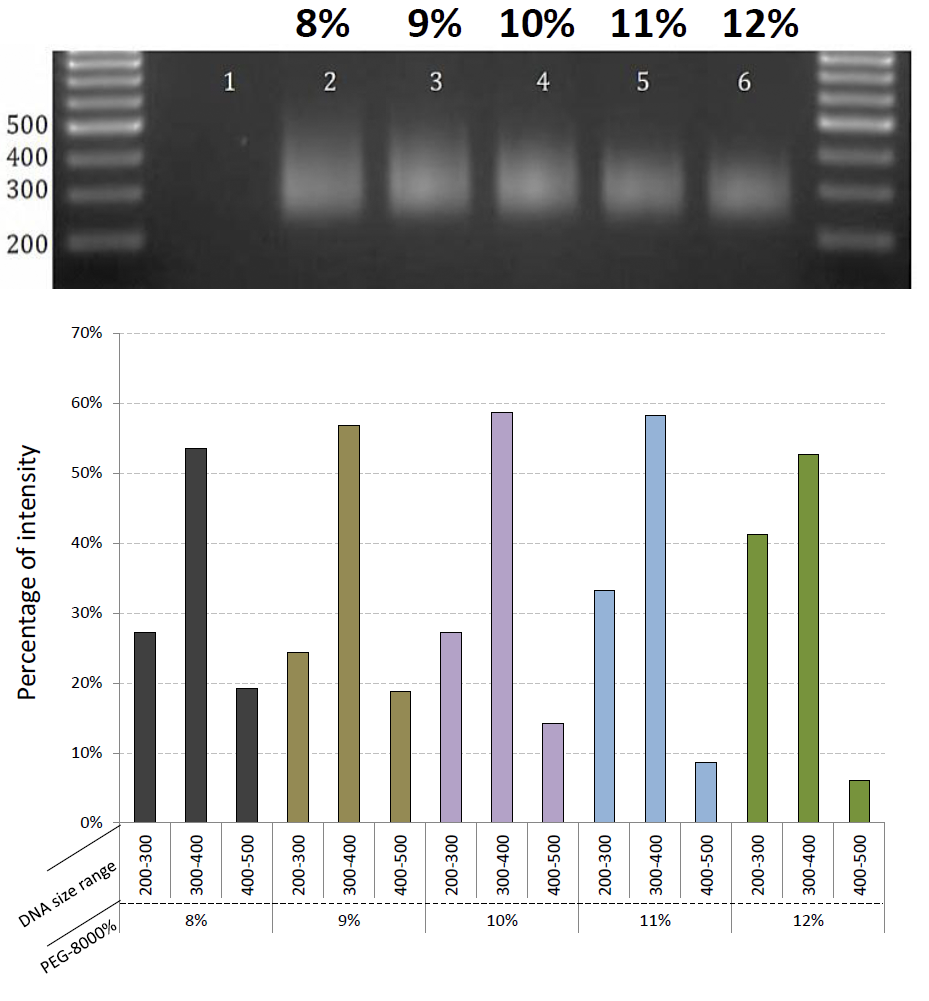

Supplement: Figure S3 — Library size selection using modified SPRI buffer. Top shows the EtBr-stained gel image of final library sizes using different concentrations of PEG-8000 in the modified buffer. Lane 1 is the control without any input DNA. Lanes 2–6 show results of using 8%–12% PEG-8000 in the buffer. Bottom Image shows ImageJ analysis result of the intensity distribution of the gel image. (TIF) [file pone.0026426.s003.tif]

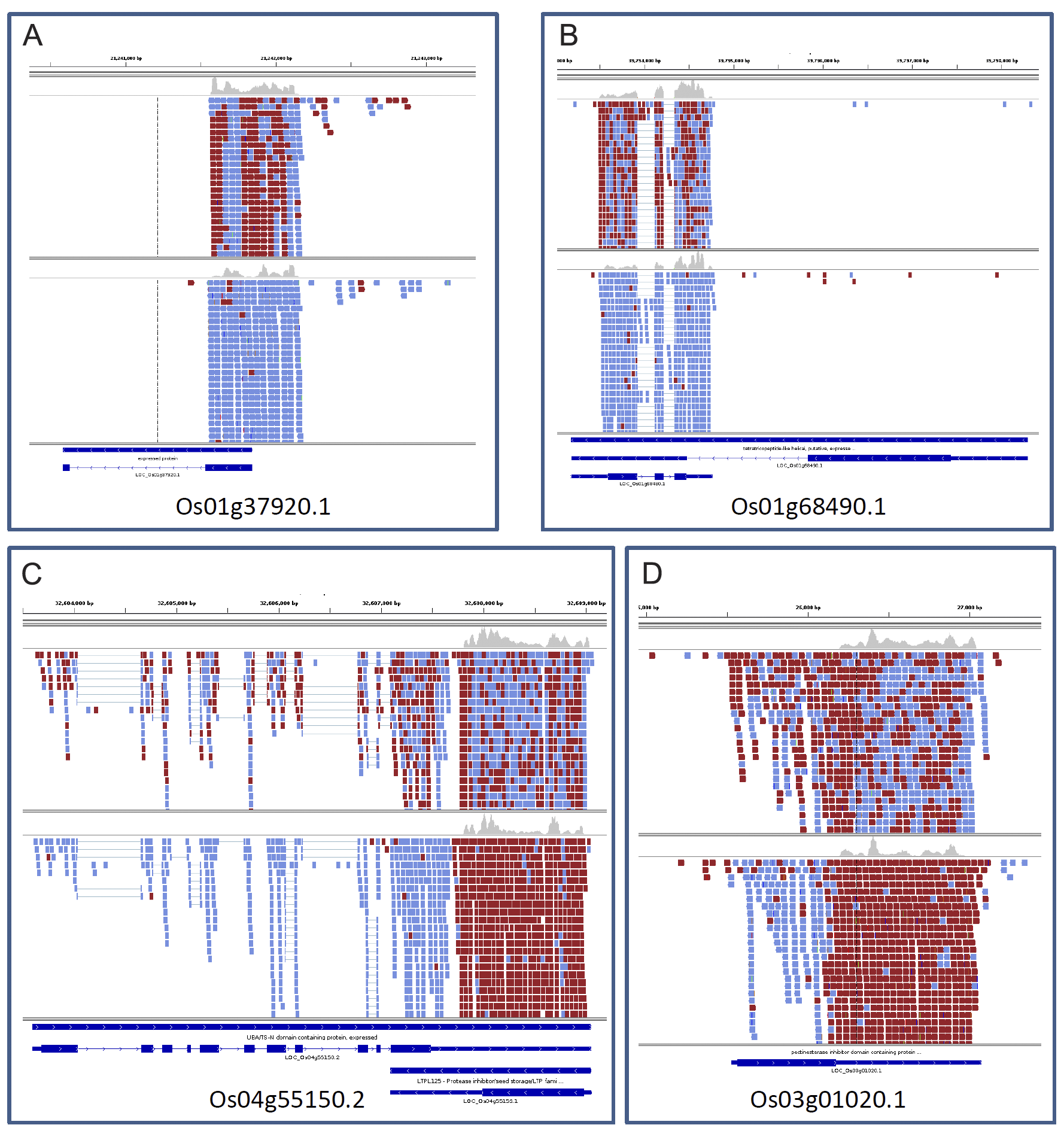

Supplement: Figure S4 — Examples of read alignments from NSS and SS RNA-seq methods. The four images display alignments visualized using IGV for rice gene model (a) Os01g37920.1, (b) Os01g68490.1, (c) Os03g01020.1 and (d) Os04g55150.2. Each panel shows the genomic region, NSS read alignment, SS read alignments, and gene models. Red and blue colors designate the directionality of reads. (TIF) [file pone.0026426.s004.tif]

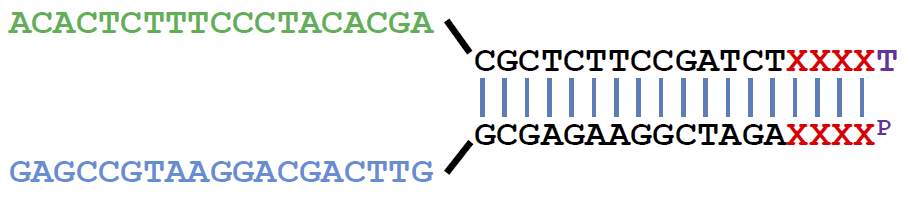

Supplement: Figure S5 — Schematic of multiplexing adaptor. Green and blue nucleotides represent the non-complementary arms. Black shows the paired region, and red represents the index sequences. Purple T is the non-paired overhang at the 3′-end and p stands for phosphorylation at 5′-end. (TIF) [file pone.0026426.s005.tif]

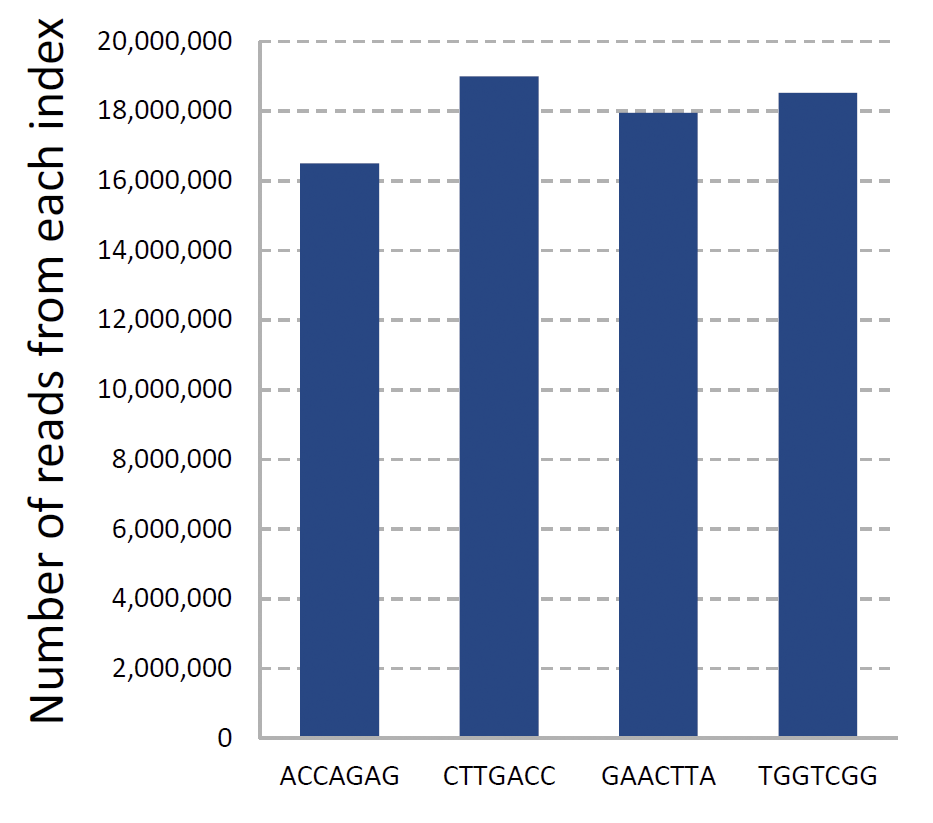

Supplement: Figure S6 — Read distribution from 7-bp indices on the HiSeq2000 platform. Each bar represents the absolute number of deconvoluted reads from one lane of HiSeq200. (TIF) [file pone.0026426.s006.tif]

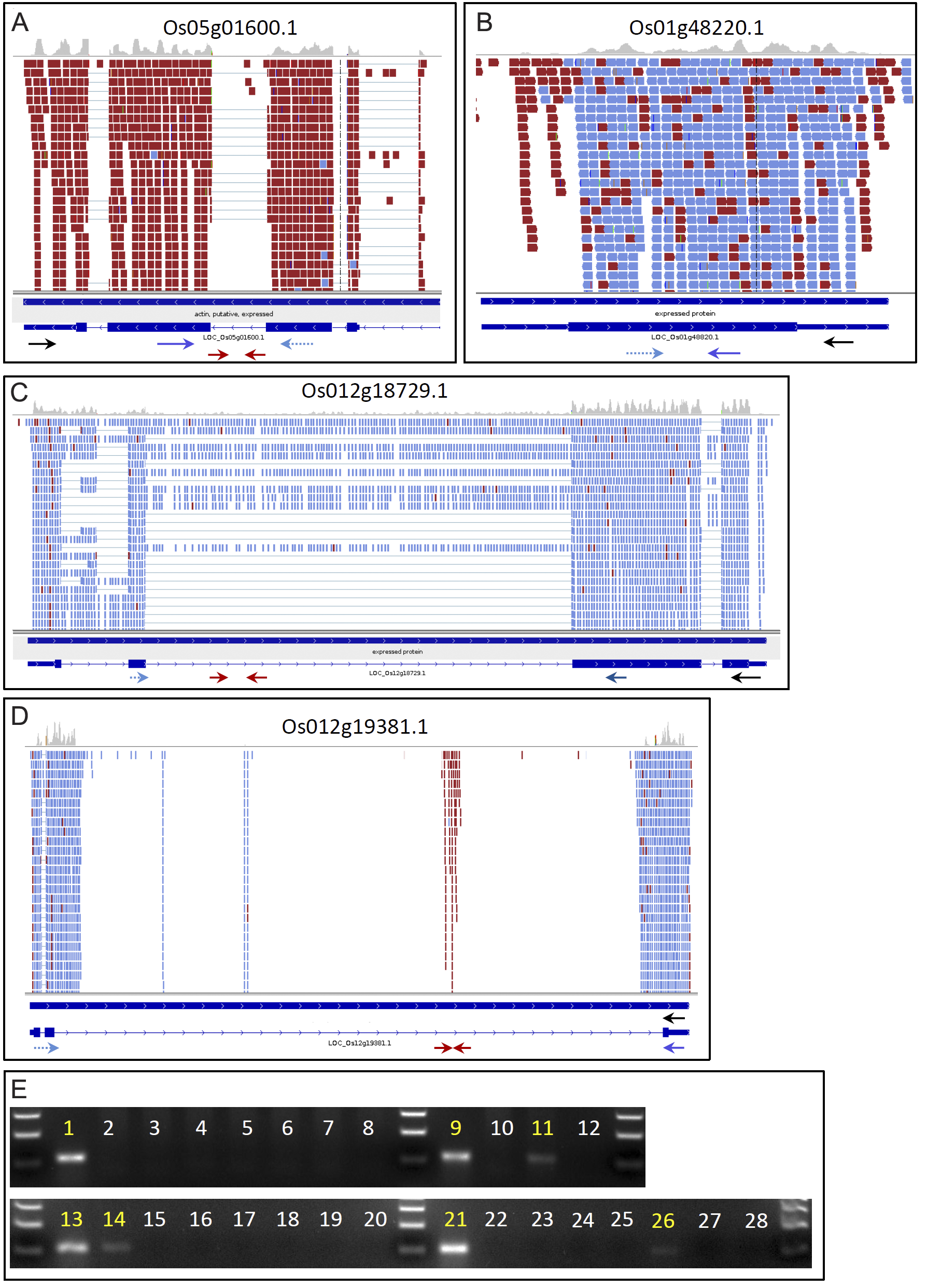

Supplement: Figure S7 — IGV visualization and RT-PCR verification of significantly expressed anti-sense transcripts and introns. (A–D) Screenshots of IGV showing (A) Os0501600.1 (Actin), (B) Os01g48220.1, (C) Os012g18729.1 and (D) Os012g19381.1. Red arrows indicate the primer pairs used to detect intronic expression. Blue arrows indicate the primers used for directional exonic expression detection, and the dashed primers were also used as the gene-specific primer for antisense first-strand cDNA synthesis. The black arrow indicates the gene-specific primer for sense first-strand cDNA synthesis. (E) Gel images of the RT-PCR results showing the existence of intronic and anti-sense expression. Lanes 1–8 are the results of amplification from Os05g01600.1 as follows: lane 1 sense exon primers(blue) using sense 1st strand cDNA (black); Lane 2 intron primers (red) with sense 1st strand template; lane 3 and 4, – RT negative control of lane 1 and 2; lane 5, exon primer (blue) with anti-sense first-strand cDNA as template (dashed blue primer); Lane 6, intron primers (red) with anti-sense 1st strand template; lane 7 and 8, - RT negative control of lane 5 and 6. Lane 13–20 (Os12g18729.1) and lane 21–28 (Os012g19381.1) follow the exact format of lane 1–8. Lane 9 and 11 shows sense and anti-sense detection of exonic expression of Os01g48220.1, while lane 10 and 12 are the –RT negative controls of lane 9 and 10 respectively. Lane number colored yellow indicate presence of amplified PCR product. DNA ladder of 100, 200 and 300 bps are not labeled with numbers. (TIF) [file pone.0026426.s007.tif]

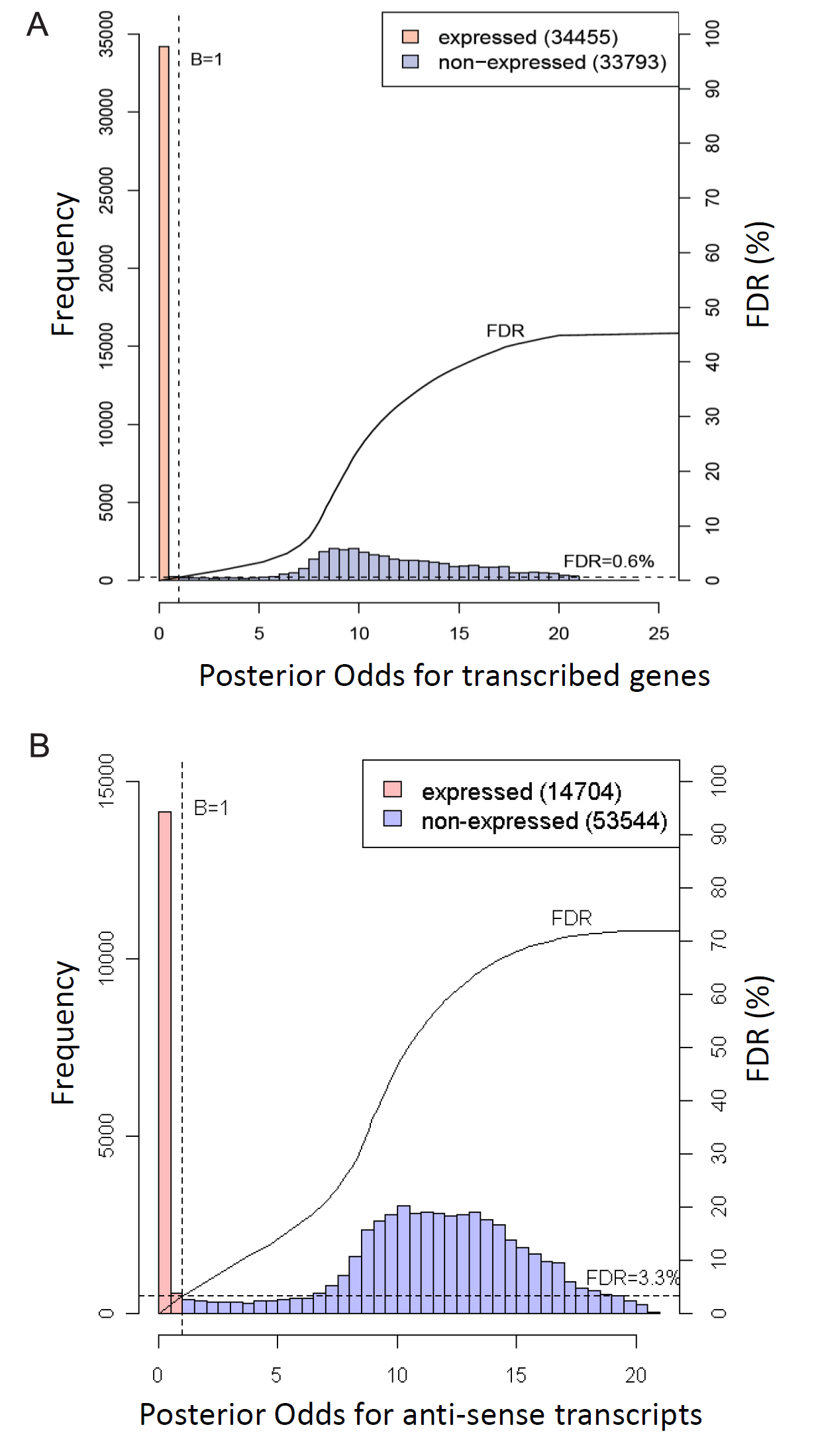

Supplement: Figure S8 — Detection of significantly expressed transcribed genes and anti-sense transcripts. The plots show the posterior odds distribution, B, and corresponding FDR at each cutoff of the posterior odds for (a) transcribed genes and (b) anti-sense transcripts. At the cutoff values of 1 for posterior odds, the associated FDR levels were estimated to be 0.6% and 3.3% for the transcribed genes and anti-sense transcripts, respectively. (TIF) [file pone.0026426.s008.tif]

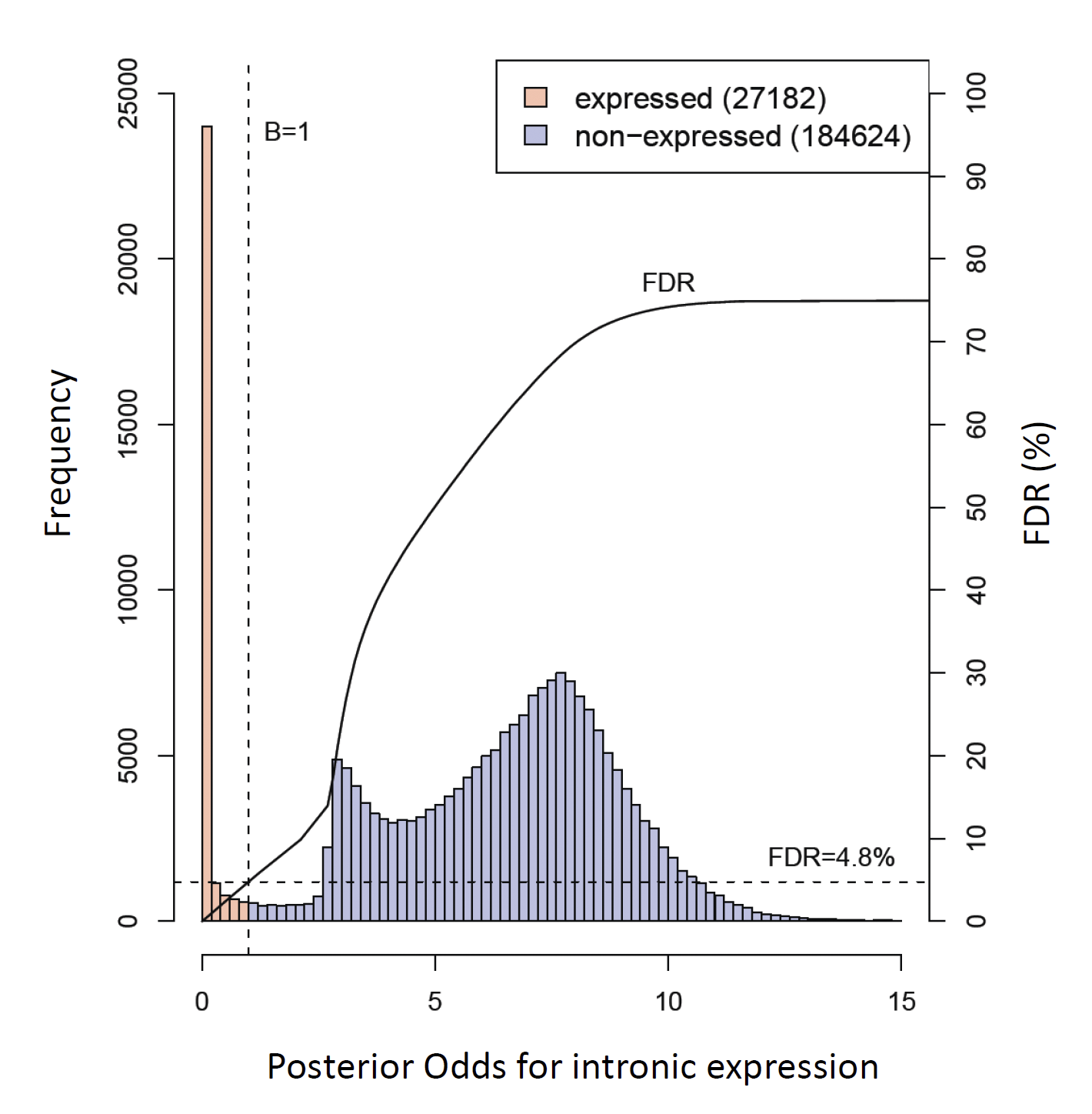

Supplement: Figure S9 — Detection of significantly expressed introns. The plot shows the distributions of posterior odds and corresponding FDR at each cutoff of the posterior odds. The cutoff value for posterior odds of B = 1 corresponds to an FDR level of approximately 4.8%. (TIF) [file pone.0026426.s009.tif]
