# Supplementary material for: A Low-Cost Library Construction Protocol and Data Analysis Pipeline for Illumina-Based Strand-Specific Multiplex RNA-Seq
Source: PLoS One. 2011 Oct 19;6(10):e26426. doi: 10.1371/journal.pone.0026426 (PMC3198403; doi:10.1371/journal.pone.0026426)
Supplement: File S1 — Protocol for constructing of strand-specific multiplex RNA-seq libraries for Illumina platforms in PDF format. (PDF) [file pone.0026426.s015.pdf]

Multiplexed Strand-specific RNA-Seq Library Preparation for Illumina Sequencing Platforms

Important Things to know before you start:

- This protocol generates strand-specific reads, but may lead to slightly reduced coverage at 5'-end of mRNA transcripts if used for single-end sequencing. Using paired-end sequencing may resolve this limitation. (This protocol is compatible with Illumina paired-end sequencing flowcell and the most recent Hiseq2000 platform.)

- This protocol is suitable with various amount of mRNA input (100 to 0.5ng). When a high amount of input is used, the number of PCR cycles needs to be reduced for the final amplification. Optimization is required for starting material amount and PCR cycles.

- The fragmentation step should be optimized in your own lab. You will want to start with RNA fragments that peak at around 200 bps.

- We have access to 15 indexed Y-adaptors, which enables one to multiplex 15 samples in one lane without observed index biases. This design has the potential to be expanded to higher numbers of indices. However, the most recent Hiseq2000 platform is not very compatible with our current adaptors. A work-around is to spike in 10% Phix control reads into each lane, but we are working on new designs that will be compatible with HiSeq2000 platform.

- All procedures can be carried out in 100ul thin-wall PCR tubes (expect for the mRNA purification steps) to save time and increase throughput. We use a custom-made magnetic stand for the PCR tubes.

- It is strongly encouraged to preform mRNA purification and dsDNA production in one day. Do not use mRNA samples stored in -80C longer than 2 weeks.

Things to be aware when working with SPRI beads

- When using RNA SPRI beads, adding half volume of 100% of ETOH helps with recovery of RNA fragments that are smaller than 100 bps. Adding PEG-8k also helps.

- When not being used, the SPRI bead suspension should be kept at 4°C or on ice. Every time, right before use, votex the suspension and make sure the beads are well mixed. Do this whenever you need to aliquot beads from stock tubes also, even when the bead suspension looks uniform.

- Do not over-dry SPRI beads, as this leads to difficult re-hydration and elution. If beads remain black chunks in the elution water, continue to vortex and make sure the elution mixture is uniform. Also increase the elution time to 5 minutes when this happens (usually 2 minutes).

- Elution efficiency with water is very high, there is no need to elute SPRI beads more than once, as this will not increase your yield but rather dilute your elute concentration.

Protocol 1: Preparation of mRNA fragments

1.1 mRNA purification

1. Use purified RNA (2-15 µg) and top with Nuclease-free H_2O to 50 µL.
2. Heat RNA sample at 65°C for 2 min (heat plate) and immediately chill on ice before use.
3. Wash 40 µL Dynabeads Olgio (dT)25 twice with 100 µL Binding Buffer and afterwards suspend washed Dynabeads in 50 µL of binding buffer.
   - *If processing more than one samples, this step can be combined: e.g. wash 320 µL beads at once for 8 samples.*
   - *You can use as low as 15 µL Dynabeads for this step to save costs*
4. Add 50 µL of Dynabeads to the 50 µL RNA, incubate with vortexing at RT for 10 min.
5. Put on magnetic stands (wait until the liquid is clear and beads are pulled to the side of the tube) and then remove liquid without disturbing beads.
6. Take tubes off the magnetic stands and wash Dynabeads twice with 150 µL of Washing Buffer.
7. Add 50 µL Elution buffer to washed and reasonably dry Dynabeads to elute the mRNA.
8. Place tubes onto 80 °C hot incubator/shaker for 2 min with mild agitation.
9. Immediately place on the magnet collector and let the liquid clear.
10. Pipette the eluted 50 µL mRNA to a new Lo-bind® tube and keep on ice.
11. Wash the original beads twice with 120 µL of water, and suspend in 50 µL of binding buffer after the 2nd wash.
12. Mix the 50 µL washed beads to the 50 µL mRNA elute in the new tube.
13. Mix well and incubate at RT for 10 min with vortexing.
14. Wash twice with 150 µL of Washing Buffer
15. Add 16 µL Elution buffer and heat at 80 °C on incubator/shaker for 2 min with mild agitation to elute the mRNA from the beads.

1.2 mRNA fragmentation

1. Assemble the following mix
    - 5x First Strand Buffer 4 µL
    - mRNA (50 ng or variable) x µL
    - Spiking RNA (optional) y µL
    - Nuclease free water (8-x-y) µL
2. Incubate at 94°C for 5 min, put the tubes on ice
    - *Incubation time should be experimentally determined in your own lab. Your fragmented mRNA should peak around 200-250 bps.*

Protocol 2: cDNA synthesis

2.1 Reverse Transcription

1. Assemble the following mix
    - mRNA 12 µL
    - Random Primer 0.5 µL
    - SupeRase-In 0.75 µL
    - DTT (100 mM) 1 µL
2. Heat at 65 °C for 3 min, place on ice and add
    - Nuclease free water 4 µL
    - DTT (100 mM) 1 µL
    - dNTPs (25 mM) 0.1 µL
    - SupeRase-In 0.5 µL
    - M-MuLV Reverse Transcriptase 0.5 µL
3. Perform reverse transcription reaction using following PCR program:
    - 25°C 10min
    - 42°C 50min
    - 70°C 15min
    - 4 °C Hold
4. Purify the RNA/cDNA hybrid using 38 µL RNAClean XP with 19 µL ethanol (100%).
5. Elute mRNA with 16 µL Nuclease Free H_2O and transfer to a new tubes

2.2 Second-strand synthesis with dUTPs

1. Prepare the 2nd strand reaction on ice as follow:
   - RNA/cDNA hybrid 16 µL
   - 10x Blue Buffer (or NEB buffer 2) 2 µL
   - dUTP mix (10mM dA, dC, dG and 20 mM dU) 1 µL
   - RNase H 0.5 µL
   - DNA polymerase I 1 µL
   - DTT (100mM) 0.5 µL
2. Incubate at 16°C for 2.5 hours
3. Purify *ds*DNA using 38 µL of AMPure XP beads with 19 µL ethanol (100%)
4. Elute with 32 µL ultra-pure Qiagen EB. Save 16 µL of elute in -80°C freezer with date and sample details

Protocol 3: Library construction

3.1 End-repair

1. Prepare the reaction on ice as follow:
   - *ds*DNA 16 µL
   - 10x End Repair Buffer (or NEB PNK buffer) 2 µL
   - 10 mM dNTP mix 1 µL
   - End Repair Mix LC 1 µL
2. Incubate at 20°C for 30 min in PCR machine
3. Purify end-repaired DNA using 28 µL of AMPure XP beads with 14 µL ethanol (100%)
4. Elute with 17 µL Nuclease Free H_2O

3.2 dA-tailing

1. Prepare the reaction as follow:
   - DNA 17 µL
   - 10x blue Buffer (NEB buffer 2) 2 µL
   - 10 mM dATP mix 1 µL
   - Klenow 3'-5' exo$^-$ (Enzymatics) 0.5 µL
2. Incubate at 37°C for 30 min
3. Purify A-tailed DNA using 28 µL of AMPure XP beads with 14 µL ethanol (100%)
4. Elute with 10 µL Nuclease Free H_2O

3.3 Y-shape Adapter Ligation

1. Prepare the reaction on ice as follow:
   - DNA 10 µL
   - Index PE adapters (2 uM) 1 µL
   - 2x Ligation Buffer 12 µL
   - T4 DNA Ligase 1 µL
2. Incubate at 20°C for 20 min in PCR machine
3. Take 12 µL (half) and save in -80 ºC freezer with date and sample details
4. Take the other half (12 µL) of the ligation product and mix with 12 µL of "12P XP" beads
5. Incubate at RT for 6 min, keep the supernatant (24 µL) and discard the beads
6. Mix the supernatant with 12 µL of AMPure XP beads and 5 µL 40% of PEG8000
7. Incubate at RT for 6 min, wash twice and elute in 10 µL Nuclease Free H_2O
8. Mix with 12 µL of AMPure XP, RT for 6 min, wash once and elute in 30 µL Qiagen EB
9. Save 15 µL of elute in -80 ºC freezer box with date and sample details

Protocol 4: Library amplification and multiplexing

4.1 dUTP excision and amplification

1. Digest 2nd strand DNA with Uracil DNA Glycosylase at 37 °C for 30 min
   - 15 µL DNA 15 µL
   - Uracil DNA Glycosylase 1 µL
2. Prepare the PCR reaction on ice as follow:
   - UNG digested DNA 16 µL
   - PE Primer (5 uM each) 2 µL
   - 5x Phusion HF Buffer 6 µL
   - 10 mM dNTP 1 µL
   - H_2O 4.5 µL
   - Phusion Hot Start 2 DNA Polymerase 1 µL
3. PCR cycle
   - 94 °C 30 sec
   - 98°C 10 sec
   - 65°C 30 sec
   - 72°C 30 sec
   - 72°C 5 min
   - 4°C

   (98°C / 65°C / 72°C steps:) **Variable Cycles**
4. Purify library using 43 µL of AMPure XP beads
5. Elute with 12 µL Qiagen EB

4.2 Prepare indexed library

1. Measure each indexed library concentration using 2 µL purified library DNA in an Invitrogen Qubit machine with the DNA HS protocol. (*Nanodrop should never be used for quantification of library, as it leads to unevenness among indexed samples*).

2. Combine equal amount of indexed libraries, depending on the concentrations of each library and the desired final volume (no less than 10 µL).
